# Occurrence of urea-based soluble epoxide hydrolase inhibitors from the plants in the order Brassicales

**DOI:** 10.1371/journal.pone.0176571

**Published:** 2017-05-04

**Authors:** Seiya Kitamura, Christophe Morisseau, Todd R. Harris, Bora Inceoglu, Bruce D. Hammock

**Affiliations:** Department of Entomology and Nematology, and UC Davis Comprehensive Cancer Center, University of California Davis, Davis, California, United States of America; Universita degli Studi di Catania, ITALY

## Abstract

Recently, dibenzylurea-based potent soluble epoxide hydrolase (sEH) inhibitors were identified in *Pentadiplandra brazzeana*, a plant in the order Brassicales. In an effort to generalize the concept, we hypothesized that plants that produce benzyl glucosinolates and corresponding isothiocyanates also produce these dibenzylurea derivatives. Our overall aim here was to examine the occurrence of urea derivatives in Brassicales, hoping to find biologically active urea derivatives from plants. First, plants in the order Brassicales were analyzed for the presence of 1, 3-dibenzylurea (compound **1**), showing that three additional plants in the order Brassicales produce the urea derivatives. Based on the hypothesis, three dibenzylurea derivatives with sEH inhibitory activity were isolated from maca (*Lepidium meyenii*) roots. Topical application of one of the identified compounds (compound **3**, human sEH IC_50_ = 222 nM) effectively reduced pain in rat inflammatory pain model, and this compound was bioavailable after oral administration in mice. The biosynthetic pathway of these urea derivatives was investigated using papaya (*Carica papaya*) seed as a model system. Finally, a small collection of plants from the Brassicales order was grown, collected, extracted and screened for sEH inhibitory activity. Results show that several plants of the Brassicales order could be potential sources of urea-based sEH inhibitors.

## Introduction

Soluble epoxide hydrolase (sEH, EC 3.3.2.10) is the major enzyme responsible for the hydrolysis of epoxy fatty acids to their corresponding diols in humans and other mammals [1]. These epoxy fatty acids are pleiotropic endogenous mediators with key functions in inflammation, pain and blood pressure regulation [1–3]. Increasing the levels of endogenous epoxy fatty acids by inhibiting sEH has been shown to block and resolve inflammation, reduce pain and lower blood pressure in numerous *in vivo* animal models [2,4–9]. The 1, 3-disubstituted urea moiety is known as a pharmacophore of many potent sEH inhibitors, in which the urea mimics both the epoxide substrate and the transition state of epoxide hydrolysis, leading to competitive inhibition of sEH [10–13]. Several groups are working to move sEH inhibitors to the clinic for treating human and equine disorders [14]. So far these compounds appear to have a large therapeutic index and thus provide an excellent margin of safety [1]. However, this traditional process of drug development takes many years, and none of sEH inhibitors are in clinical use yet. Alternatively, sEH inhibitors derived from natural products, especially edible vegetables, could provide a shorter path to treating patients and companion animals, offering inexpensive therapeutics to patients that will not require the same regulatory barriers as pharmaceuticals [15,16]. In addition, study of these natural products will explain the modes of action of some natural remedies.

**Fig 1:**
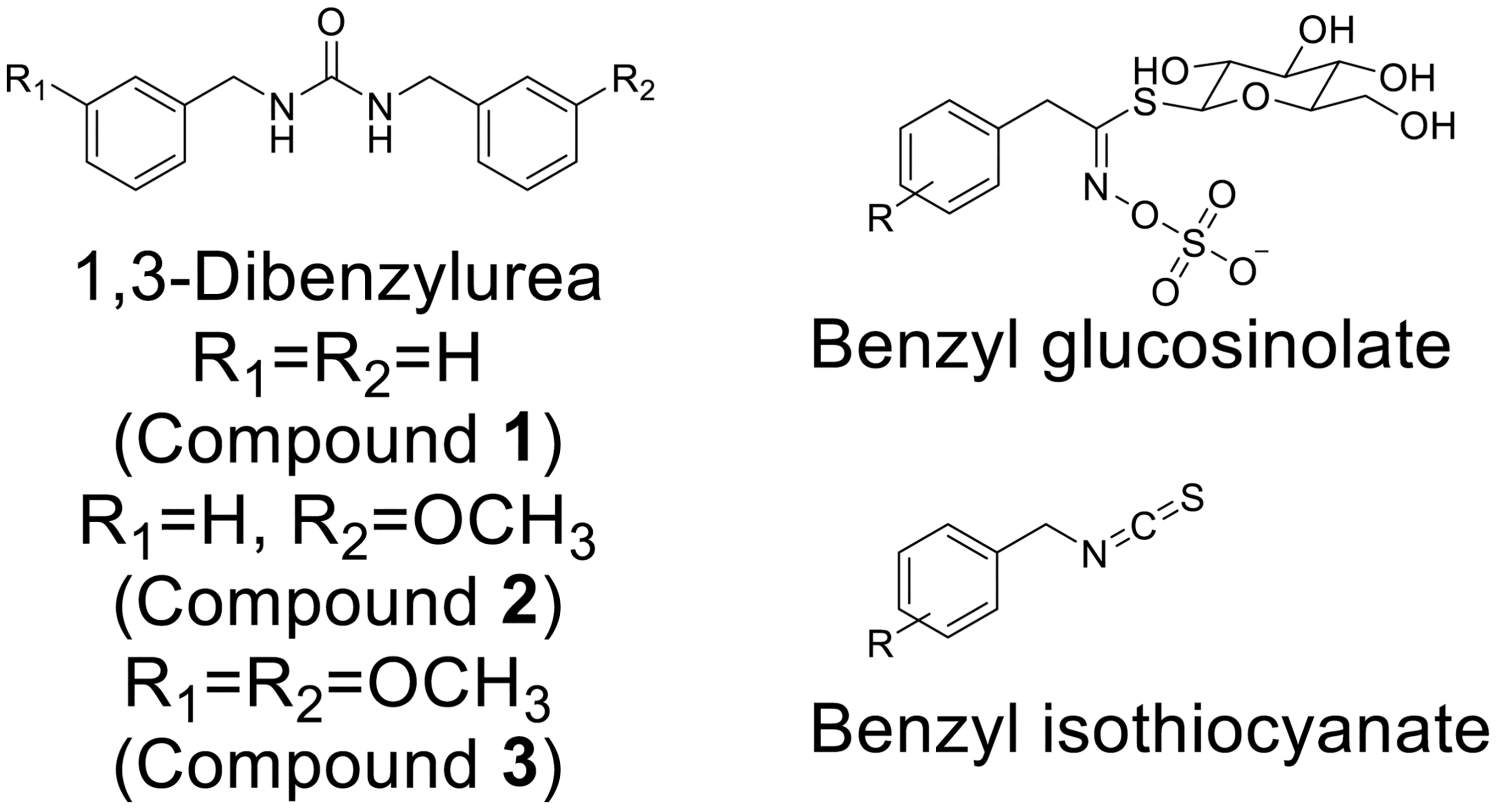
Chemical structures of 1, 3-dibenzylurea, benzyl glucosinolate and isothiocyante.

Tsopmo *et al*. reported 1, 3-dibenzylurea derivatives in the root of a plant in Cameroon, *Pentadiplandra brazzeana*, and we recently reported these urea derivatives as potent sEH inhibitors [17,18]. A few others also reported benzylurea derivatives in plants [19–21]. Interestingly, all of these plants are members of the order Brassicales. These plants were also reported to produce benzyl glucosinolates or benzyl isothiocyanates (chemical structures shown in Fig 1) [22–25]. Glucosinolates and isothiocyanates are well-recognized secondary metabolites of plants in the order Brassicales, while urea derivatives in nature have not been studied extensively. We hypothesized that some of the plants that produce benzyl glucosinolates and benzyl isothiocyanates also produce these dibenzylurea derivatives.

Our overall aim was to examine the occurrence of urea derivatives in Brassicales, hoping to find biologically active urea-based sEH inhibitors from plants. To accomplish this, we looked for 1, 3-dibenzylurea (compound **1**) in plants in the order Brassicales that have been reported to produce benzyl glucosinolate or benzyl isothiocyanate. Next, *meta* methoxy substituted benzylurea derivatives, which were predicted based on the hypothesis, were isolated from maca (*Lepidium meyenii*) root and assayed for sEH inhibitory potency *in vitro*. The most potent compound (compound **3**) showed *in vivo* analgesic effects in a rat inflammatory pain model, and was bioavailable after oral administration. Possible biosynthetic pathways of compound **1** were studied using papaya seed as a model system. Finally, a small collection of plants from the Brassicales order was grown, collected, extracted and screened for sEH inhibitory activity and for the occurrence of urea derivatives.

## Materials and methods

### General experimental procedures

All reagents and solvents were purchased from commercial suppliers and were used without further purification. All reactions were performed in an inert atmosphere of dry nitrogen or argon. UV absorption spectra were measured on a Varian Cary 100 Bio UV-Visible Spectrophotometer. Melting points were determined using an OptiMelt melting point apparatus. NMR spectra were collected using a Varian 400 or 600 MHz, or Bruker Avance III 800 MHz spectrometer with chemical shifts reported relative to residual deuterated solvent peaks or a tetramethylsilane internal standard. Accurate masses were measured using a LTQ orbitrap hybrid mass spectrometer or Micromass LCT ESI-TOF-MS. FT-IR spectra were recorded on a Thermo Scientific NICOLET IR100 FT-IR spectrometer. The purity of all synthetic compounds were found to be > 95% based on NMR analysis. The purity of the compounds that were tested in the assay were further determined by reverse phase HPLC-DAD and found to be > 95% at 254 nm absorption (LC method detailed in S3 Table).

### Plant samples

The plant species were authenticated by a botanist Dr. Ellen Dean at UC Davis Center for Plant Diversity, where a voucher specimen of papaya (*Carica papaya*) fruit (DAV211613), papaya seed (DAV211614), garden cress (*Lepidium sativum*, DAV211612, DAV211953), and water cress (*Nasturtium officinale*, DAV216312) were deposited. Fresh papaya fruit imported from Mexico was purchased at La Superior Supermercados (Woodland, CA) between Nov. 2014 and Apr. 2015. Fresh water cress produced in California was purchased at Nugget Market (Davis, CA) between Feb. 2016 and Aug. 2016. Garden cress and water cress seeds were purchased from Botanical Interests Inc. and germinated following the manufacturer’s protocol. The whole sprouts were harvested 2 weeks after seeding between Dec. 2014 and Aug. 2016. Maca dried root powder imported from Peru was purchased from Herbs America (100% Raw Maca Powder, product name ‘Maca Magic^™^’). The organism of origin was specified by DNA sequencing, and is included in the Supporting information (S1 Text). The powder specimen was deposited at the Center for Plant Diversity at UC Davis (DAV211952).

### Extraction for LC-MS/MS analysis

Fresh papaya seeds (2 g) or fresh garden cress sprouts (300 mg) were ground and incubated overnight at RT, then extracted with dichloromethane (DCM)-methanol (MeOH) (1:1) by sonication (2 x 3 mL) at RT (5 min per extraction). The dried root powder of maca (100 mg) was extracted with the same method. The extracts were concentrated *in vacuo*, reconstituted in MeOH and filtered, then diluted to the appropriate concentration for the LC-MS/MS analysis. Standard addition and recovery experiments were performed as described previously [17].

### LC-MS/MS analysis

Compounds were analyzed using a Waters Quattro Premier triple quadrupole tandem mass spectrometer (Micromass, Manchester, UK) interfaced to an electrospray ionization (ESI) source. The ESI was performed following HPLC in the positive mode at 2.51 kV capillary voltage. The source and the desolvation temperatures were set at 120 and 300°C, respectively. Cone gas (N_2_) and desolvation gas (N_2_) were maintained at flow rates of 10 and 700 L/h, respectively. Optimized conditions for mass spectrometry are shown in S1 Table. Dwell time was set to 0.1 s. A regression curve for each compound was obtained from at least six different concentrations of standard stock solutions (R^2^ > 0.99). 1, 3-Diphenylurea was used as an internal standard and was added just before the analysis. The final concentration of 1, 3-diphenylurea was adjusted to 100 nM.

The MS was coupled with a Waters Acquity UPLC (Waters, Milford, MA, USA). A Varian Pursuit5 C18 RP HPLC column (150 mm × 2.1 mm, particle size 5 μm) was used to separate the analytes. The HPLC solvent gradient is shown in S3 Table.

### Isolation and purification of urea derivatives

The dried root powder of maca (20 kg) was extracted with DCM-MeOH (1:1) by shaking (3 x 40 L) at room temperature (at least 120 min per shaking). The combined extracts were concentrated *in vacuo*, and the residue was partitioned between DCM and brine. Concentrating the DCM layer *in vacuo* yielded the crude extract (612 g) as a dark brown oil. Flash column chromatography on a Si gel column eluting with hexane: ethyl acetate (1:1) or DCM: MeOH (30:1 or 50:1) was repeated, followed by repetitive preparative scale normal phase HPLC (Phenomenex Luna Silica (2) column, 250 × 21.2 mm, 5 μm, Waters ELSD 2424 evaporative light scattering detector and 1525 Binary HPLC Pump) eluting with hexane: isopropanol (9:1) at a flow rate of 20 mL/min. Recrystallization from DCM/hexane afforded compound **1** (31 mg) and compound **2** (36 mg). Further purification by reverse phase HPLC (Phenomenex Luna C18 (2) column, 250 × 21.2 mm, 5 μm) eluting with water: MeOH (50–80% gradient in 20 min, 12 mL/min) followed by a short flash column chromatography on a Si gel eluting with DCM: MeOH (30:1) afforded compound **3** (1.5 mg). It should be noted that dibenzyl thioureas were not observed in dried maca root powder. Therefore, it is unlikely that urea derivatives in maca root were produced during the extraction and purification.

**1, 3-Dibenzylurea (compound 1)**: off-white powder (DCM); mp 166–170°C (lit.[18] 168–170°C); UV (acetonitrile) λ_max_ (log ε): 258 (2.26) nm; IR (neat) ν_max_ 3321, 1626, 1572, 1493, 1453, 1254, 752 cm^-1^; ^1^H NMR (800 MHz, DMSO-*d*_6_) δ 7.31 (t, *J* = 7.6 Hz, 4H, H-5, H-7), 7.25 (d, *J* = 6.7 Hz, 4H, H-4, H-8), 7.22 (t, *J* = 7.2 Hz, 2H, H-6), 6.44 (s, 2H, NH), 4.23 (d, *J* = 6.0 Hz, 4H, H-2). ^13^C: NMR (201 MHz, DMSO-*d*_6_) δ 158.1 (C, C-1), 140.9 (C, C-3), 128.2 (CH, C-5, C-7), 127.0 (CH, C-4, C-8), 126.6 (CH, C-6), 43.0 (CH_2_, C-2). HRESIMS *m/z* 241.1336 (S4 Fig Calculated for [C_15_H_17_N_2_O]^+^, 241.1335).

**Fig 2:**
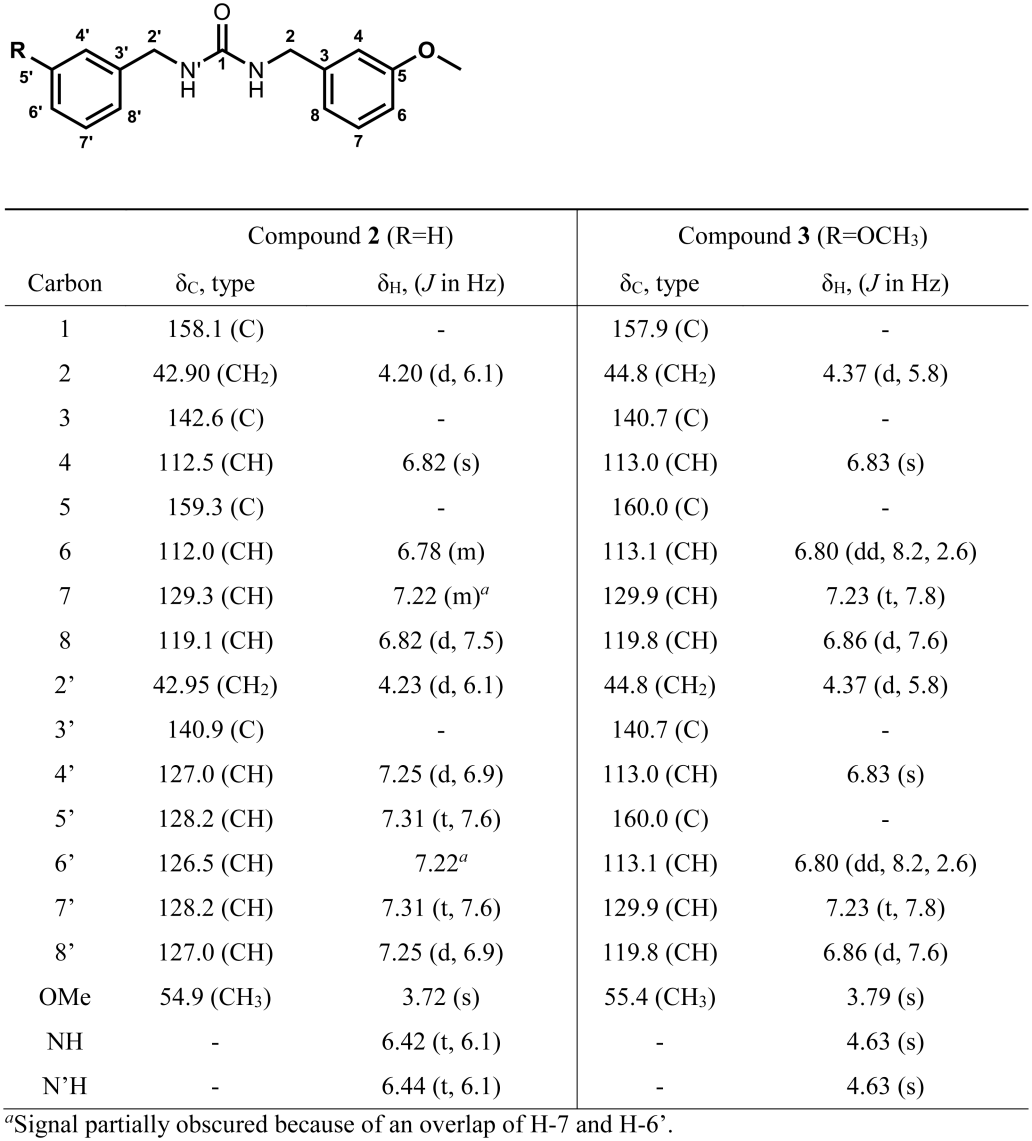
NMR spectroscopic data (^1^H 800 MHz, ^13^C 201 MHz) for compound 2 (DMSO-*d*_6_) and compound 3 (CDCl_3_).

**1-Benzyl-3-(3-methoxybenzyl) urea (compound 2)**: off-white powder (DCM); mp 101–107°C (synthetic standard (acetone) 108.3–109.1 (108.6°C); UV (acetonitrile) λ_max_ (log ε): 272 (3.25) nm; IR (neat) ν_max_ 3349, 3317, 3032, 2923, 1625, 1577, 1511, 1242, 1031 cm^-1^; ^1^H and ^13^C NMR see Fig 2. HRESIMS *m/z* 271.1441 (S5 Fig Calculated for [C_16_H_19_N_2_O_2_]^+^, 271.1441).

**1, 3-Bis (3-methoxybenzyl) urea (compound 3)**: off-white solid; UV (acetonitrile) λ_max_ (log ε): 273 (3.31) nm; IR (neat) ν_max_ 3321, 1621, 1589, 1574, 1488, 1472, 1435, 1296, 1263, 1155, 1049, 785 cm^-1^; ^1^H and ^13^C NMR see Fig 2. HRESIMS *m/z* 301.1540 (S6 Fig Calculated for [C_17_H_21_N_2_O_3_]^+^, 301.1546).

### Synthesis of ureas and thioureas

Compound **1**, 1-(adamantan-1-yl)-3-(5-(2-(2-ethoxyethoxy) ethoxy) pentyl) urea (AEPU), and 1-trifluoromethoxyphenyl-3-(1-propionylpiperidin-4-yl) urea (TPPU) were previously synthesized [12,26,27].

#### General procedure of urea and thiourea synthesis

An amine (1 equiv) was added to a solution of benzyl isocyanate or benzyl isothiocyanate in THF. After stirring for 10 min at room temperature, hexane was added and the resulting white crystals were collected. Recrystallization from acetone was repeated until the target compound was > 95% pure as judged by NMR analysis.

**1-Benzyl-3-(3-methoxybenzyl) urea (compound 2)**: off-white powder (260 mg, 0.963 mmol, 75%); mp 108.3–109.1 (108.6°C; ^1^H and ^13^C NMR: identical to compound **2** isolated from maca (Fig 2); ESI-MS [M+Na]^+^ *m/z* 293.11 (calculated for C_16_H_18_N_2_NaO_2_ 293.13), Purity > 99% (HPLC-UV (254 nm), ^*t*^R = 9.18 min).

**1, 3-Bis (3-methoxybenzyl) urea (compound 3)**: white powder (45 mg, 0.15 mmol, 15%); mp 112.6–113.8 (113.0°C; ^1^H and ^13^C NMR: identical to compound **3** isolated from maca (Fig 2); ESI-MS [M+Na]^+^ *m/z* 323.11 (calculated for C_17_H_20_N_2_NaO_3_ 323.14), Purity > 99% (HPLC-UV (254 nm), ^*t*^R = 9.01 min).

**1-Benzyl-3-(4-isopropylphenyl) urea (compound 4)**: white powder (20 mg, 75 μmol, 4%); mp 111.8–112.2 (112.0°C; ^1^H NMR (600 MHz, DMSO-*d*_6_) δ 8.43 (s, 1H), 7.34–7.29 (m, 6H), 7.25–7.22 (m, 1H), 7.08 (d, *J* = 8.3 Hz, 2H), 6.54 (t, *J* = 6.0 Hz, 1H), 4.28 (d, *J* = 5.9 Hz, 2H), 2.79 (hept, *J* = 7.0 Hz, 1H), 1.16 (d, *J* = 6.9 Hz, 6H). ^13^C NMR (151 MHz, DMSO-*d*_6_) δ 155.3, 141.1, 140.4, 138.2, 128.3, 127.1, 126.7, 126.3, 117.9, 42.7, 32.7, 24.1. ESI-MS [M+Na]^+^ *m/z* 291.15 (calculated for C_17_H_20_N_2_NaO 291.15).

**1-Benzyl-3-(4-isopropylphenyl) thiourea (compound 5)**: white powder (109 mg, 384 μmol, 38%) mp 110.0–110.3 (110.1°C; ^1^H NMR (600 MHz, DMSO-*d*_6_) δ 9.53 (s, 1H), 8.08 (s, 1H), 7.34–7.19 (m, 9H), 4.73 (d, *J* = 5.8 Hz, 2H), 2.86 (hept, *J* = 6.9 Hz, 1H), 1.19 (d, *J* = 6.9 Hz, 6H).^13^C NMR (151 MHz, DMSO-*d*_6_) δ 180.7, 144.6, 139.2, 136.7, 128.2, 127.4, 126.8, 126.5, 123.7, 47.2, 33.0, 23.9. ESI-MS [M+Na]^+^ *m/z* 307.10 (calculated for C_17_H_20_N_2_NaS 307.12).

**1, 3-Diphenethylurea (compound 8)**: white powder (15 mg, 56 μmol, 3%) mp 138.5–138.9 (138.8°C;^1^H NMR (600 MHz, DMSO-*d*_6_) δ 7.29 (t, *J* = 7.5 Hz, 4H), 7.19 (d, *J* = 8.2 Hz, 4H), 5.87 (t, *J* = 5.8 Hz, 2H), 3.21 (m, 4H), 2.66 (t, *J* = 7.2 Hz, 4H). ^13^C NMR (151 MHz, DMSO-*d*_6_) δ 157.8, 139.7, 128.6, 128.2, 125.9, 40.8, 36.14. HRESI-MS [M+H]^+^ *m/z* 269.1649 (calculated for C_17_H_21_N_2_O 269.1648). Purity 98% (HPLC-UV (254 nm), ^*t*^R = 10.12 min).

**1, 3-Diphenethylthiourea (compound 9)**: off-white crystals (219 mg, 771 μmol, 39%) mp 93–96 (94°C; ^1^H NMR (600 MHz, DMSO-*d*_6_) δ 7.45 (s, 2H), 7.30 (t, *J* = 7.6 Hz, 4H), 7.25–7.16 (m, 6H), 3.59 (s, 4H), 2.79 (t, *J* = 7.6 Hz, 4H). ^13^C NMR (101 MHz, DMSO-*d*_6_) δ 182.1, 139.4, 128.6, 128.3, 126.1, 44.9, 40.1, 39.9, 39.7, 39.5, 39.3, 39.1, 38.9, 34.8. HRESI-MS [M+H]^+^ *m/z* 285.1422 (calculated for C_17_H_21_N_2_S 285.1424). Purity 98% (HPLC-UV (254 nm), ^*t*^R = 11.45 min).

**1-(4-Hydroxyphenethyl)-3-phenethylurea (compound 10)**: white powder (135 mg, 475 μmol, 48%) mp 157–159 (158°C; ^1^H NMR (600 MHz, DMSO-*d*_6_) δ 9.14 (s, 1H), 7.29 (t, *J* = 7.5 Hz, 2H), 7.22–7.17 (m, 3H), 6.97 (dd, *J* = 8.7, 2.7 Hz, 2H), 6.70–6.64 (m, 2H), 5.83 (dt, *J* = 27.7, 5.8 Hz, 2H), 3.24–3.18 (m, 2H), 3.14 (q, *J* = 6.5 Hz, 2H), 2.66 (t, *J* = 7.2 Hz, 2H), 2.54 (t, *J* = 7.3 Hz, 2H). ^13^C NMR (151 MHz, DMSO-*d*_6_) δ 157.8, 155.5, 139.7, 129.7, 129.4, 128.6, 128.2, 125.9, 115.0, 41.2, 40.8, 36.2, 35.3. HRESI-MS [M+H]^+^ *m/z* 285.1596 (calculated for C_17_H_21_N_2_O_2_ 285.1598). Purity >99% (HPLC-UV (254 nm), ^*t*^R = 8.72 min).

**1-(4-Methoxybenzyl)-3-phenethylurea (compound 11)**: white powder (303 mg, 475 μmol, 53%) mp 122.8–123.4 (123.1°C; ^1^H NMR (400 MHz, DMSO-*d*_6_) δ 7.33–7.24 (m, 2H), 7.24–7.11 (m, 5H), 6.91–6.83 (m, 2H), 6.26 (t, *J* = 5.9 Hz, 1H), 5.88 (t, *J* = 5.8 Hz, 1H), 4.12 (d, *J* = 5.9 Hz, 2H), 3.72 (s, 3H), 3.30–3.19 (m, 2H), 2.68 (t, *J* = 7.2 Hz, 2H). ^13^C NMR (101 MHz, DMSO-*d*_6_) δ 158.0, 157.9, 139.7, 132.8, 128.7, 128.3, 128.3, 125.9, 113.6, 55.0, 42.3, 41.0, 36.2. HRESI-MS [M+H]^+^ *m/z* 285.1597 (calculated for C_17_H_21_N_2_O_2_ 285.1598). Purity 99% (HPLC-UV (254 nm), ^*t*^R = 9.69 min).

### General procedure of chemical demethylation

To a solution of compound **3** in dichloromethane, 1 M tribromoborane (1.2 equiv or 3.0 equiv) solution in DCM was added dropwise at -78°C. The reaction was slowly warmed up to RT, and stirred for 24 h. To this solution was added dropwise water at 0°C, then warmed to RT. To this solution was added ethyl acetate, extracted, and washed with brine twice. The organic layer was dried over MgSO_4_, filtered and concentrated *in vacuo*. The target compound was purified by column chromatography followed by recrystallization from DCM.

**1-(3-Hydroxybenzyl)-3-(3-methoxybenzyl) urea (compound 6)**: white powder (5 mg, 17 μmol, 5%) mp 124.8–125.6 (125.3°C; ^1^H NMR (600 MHz, DMSO-*d*_6_) δ 9.30 (s, 1H), 7.22 (t, *J* = 7.8 Hz, 1H), 7.08 (t, *J* = 7.7 Hz, 1H), 6.83–6.81 (m, 2H), 6.78 (dd, *J* = 8.5, 2.6 Hz, 1H), 6.66 (d, *J* = 8.3 Hz, 2H), 6.61–6.59 (m, 1H), 6.38 (dt, *J* = 11.7, 6.1 Hz, 2H), 4.20 (d, *J* = 6.0 Hz, 2H), 4.14 (d, *J* = 6.0 Hz, 2H), 3.72 (s, 3H). ^13^C NMR (151 MHz, DMSO-*d*_6_) δ 159.7, 158.4, 157.8, 143.0, 142.8, 129.7, 129.6, 119.6, 118.0, 114.3, 113.9, 112.9, 112.4, 55.4, 43.3 (x2). ESI-MS [M+H]^+^ *m/z* 287.12 (calculated for C_16_H_19_N_2_O_3_ 287.14), Purity >99% (HPLC-UV (254 nm), ^*t*^R = 7.87 min).

**1, 3-Bis(3-hydroxybenzyl)urea (compound 7)**: white powder (58 mg, 213 μmol, 64%) mp 155.3–157.0 (156.1°C; ^1^H NMR (600 MHz, DMSO-*d*_6_) δ 9.30 (s, 2H), 7.08 (t, *J* = 8.0 Hz, 2H), 6.67–6.65 (m, 4H), 6.60 (ddd, *J* = 8.0, 2.3, 1.2 Hz, 2H), 6.33 (t, *J* = 6.0 Hz, 2H), 4.14 (d, *J* = 6.0 Hz, 4H). ^13^C NMR (151 MHz, DMSO-*d*_6_) δ 158.0, 157.3, 142.3, 129.2, 117.6, 113.9, 113.5, 42.9. ESI-MS [M+H]^+^ *m/z* 273.13 (calculated for C_15_H_17_N_2_O_3_ 273.12), Purity 95% (HPLC-UV (254 nm), ^*t*^R = 5.81 min).

### Variable incubation conditions and trapping experiments

For each of the conditions, extraction and incubation was performed as described above. For the conditions without overnight incubation, DCM/MeOH (1:1) was added immediately after grinding the plant material and then extracted. For methanol treatment, MeOH (2 mL) was added and then ground and incubated at RT overnight. For microwave heating, 2 g of papaya seeds were put in a sealed tube and heated to 100°C for 20 min using a high-pressure microwave digestion unit (Ethos SEL. Milestone, Italy). After cooling to RT, seeds were ground and incubated at RT overnight. For liquid nitrogen treatment, before grinding, fresh papaya seeds were frozen by the addition of liquid nitrogen (approximately 5 mL), then ground and extracted with DCM/MeOH (1:1) without incubation. For the trapping experiments, 2 mL of an aqueous solution (containing 1% v/v DMSO) of 4-isopropylaniline (10 μM) or compound **5** (10 μM) was added to fresh papaya seeds (2 g). This was ground thoroughly and incubated overnight at RT.

### Enzyme purification

Recombinant sEH was produced in insect High Five cells using recombinant baculovirus expression vectors, and purified by affinity chromatography as reported previously [28,29]. The enzyme appeared as a single band (0.3 μg loading) with an estimated purity of more than 95% by Coomassie Brilliant Blue staining following SDS-PAGE separation. The final recombinant sEH preparations had no esterase or glutathione *S*-transferase activities, which interfere with the CMNPC assay as described below.

### Measurement of sEH inhibition by fluorescent assay (CMNPC assay)

IC_50_ values were determined as described previously [30] using cyano (2-methoxynaphthalen-6-yl) methyl *trans*-(3-phenyl-oxyran-2-yl) methyl carbonate (CMNPC) as a fluorescent substrate. Recombinant sEH (0.96 nM) was incubated with inhibitors for 5 min in 25 mM bis-Tris/HCl buffer (pH 7.0) containing 0.1 mg/mL of BSA at 30°C prior to substrate introduction ([S] = 5 μM). Activity was measured by determining the appearance of the 6-methoxy-2-naphthaldehyde with an excitation wavelength of 330 nm and an emission wavelength of 465 nm for 10 min.

### Ethics statement

All of the animal experiments were conducted in line with federal regulations and were performed according to protocols approved by the Animal Use and Care Committee of the University of California, Davis.

### Nociceptive assay using the rat inflammatory pain model

Male Sprague-Dawley rats weighing 225–250 g were obtained from Charles River Laboratories and maintained in the UC Davis animal housing facility with ad libitum water and food on a 12 h/12 h light-dark cycle. Behavioral nociceptive testing was conducted by assessing mechanical withdrawal threshold using an electronic von Frey anesthesiometer apparatus (IITC, Woodland Hills, CA) as described previously [31]. The analgesic effect of compound **3** was tested using the intraplantar carrageenan-induced local inflammatory pain model [12,31]. Following baseline measurements, carrageenan (50 μL, 1% solution of carrageenan in ddH_2_O) was administered into the plantar area of one hind paw. At 4 h post administration of carrageenan, responses were measured, and immediately after these measurement, **3** (3% in PEG400/Vanicream^™^ (Pharmaceutical Specialties, Inc. Rochester, MN) = 3:7, 6 mg of compound/paw), the synthetic sEH inhibitor triclocarban (TCC, 3%), or the vehicle (200 μL of PEG400/Vanicream^™^) was administered onto the inflamed paw by topical application [4]. The cream was thoroughly massaged across the entire hind paw surface over a 1–2 min period. Subsequently, the ability of these treatments to reduce the carrageenan-induced inflammatory pain was monitored over the course of 4 h.

### Statistical analysis

Data were analyzed using SigmaPlot 11.0 for Windows (Systat Software Inc., San Jose, CA). Kruskal-Wallis One Way ANOVA on Ranks followed by Tukey Test was performed with p values < 0.05 considered significant.

### Pharmacokinetics

Pharmacokinetic profiles of the inhibitors were determined by following the procedure described previously in literature [32,33] with some modifications. Male Swiss Webster mice (7 weeks old, 24–30 g) were purchased from Charles River Laboratories. Inhibitors were dissolved in polyethylene glycol (average molecular weight: 300) to give a clear solution for oral administration. Compounds were orally administered by gavage to mice at a dose of 10 mg/kg (compound **3** & AEPU) and 0.3 mg/kg (TPPU) in 100–140 μL of vehicle depending on animal weight. Blood (10 μL) was collected from the tail vein using a pipet tip rinsed with EDTA. The blood samples were prepared according to the methods detailed previously [32], and blood samples were analyzed by LC-MS/MS.

### Brassicales library construction

Plant samples and their sources are listed in supporting information (S4 Table). The whole sprouts were harvested 1–2 weeks after seeding depending on the growth of the sprouts. Approximately 1 g of whole sprouts were ground and incubated at RT for 2 days, and extracted as described above. Extract was reconstituted in DMSO (10 mg/mL), and IC_50_ was measured using the fluorescent-based assay using CMNPC as a substrate. For MS screening, extracts were diluted 100-fold in methanol containing 100 nM AUDA (internal standard, 12-(3-adamantan-1-yl-ureido) dodecanoic acid), filtered, and analyzed for urea derivatives listed in the supporting information (S2 Table).

## Results and discussion

### Occurrence and quantification of 1, 3-dibenzylureas in Brassicales

Plants in the order Brassicales, including maca (*Lepidium meyenii*) root, papaya (*Carica papaya*) seed, and garden cress (*Lepidium sativum*) sprout, were tested for the presence of compound **1** (1, 3-dibenzylurea) using LC-MS/MS. These plant species and parts were selected based on previous reports of the presence of benzyl glucosinolate (glucotropaeolin) or benzyl isothiocyanate [23,34,35]. Compound **1** was detected in all three plants, suggesting that the occurrence of urea derivatives is conserved among these plant species (Table 1). To test the efficiency of our extraction method, synthetic standard of compound **1** was spiked into the plant samples. The recovery percentages were above 88%, suggesting that our extraction method yields sufficient compound **1** from the plant samples.

**Table 1:** Concentration of 1, 3-dibenzylurea (compound 1) in plant samples.

	Concentration of compound 1	
	ng/g fresh weight	ng/g dry weight
Papaya seed	56 ± 11	370 ± 73
Garden cress sprout	250 ± 38	11,800 ± 1,800
Maca dried root powder	-	6,900 ± 125
*Pentadiplandra brazzeana* dried root powder^a^	-	1,900 ± 200

Mean ± SD values are shown.

^*a*^Data from Kitamura *et al*., (2015).

**Fig 3:**
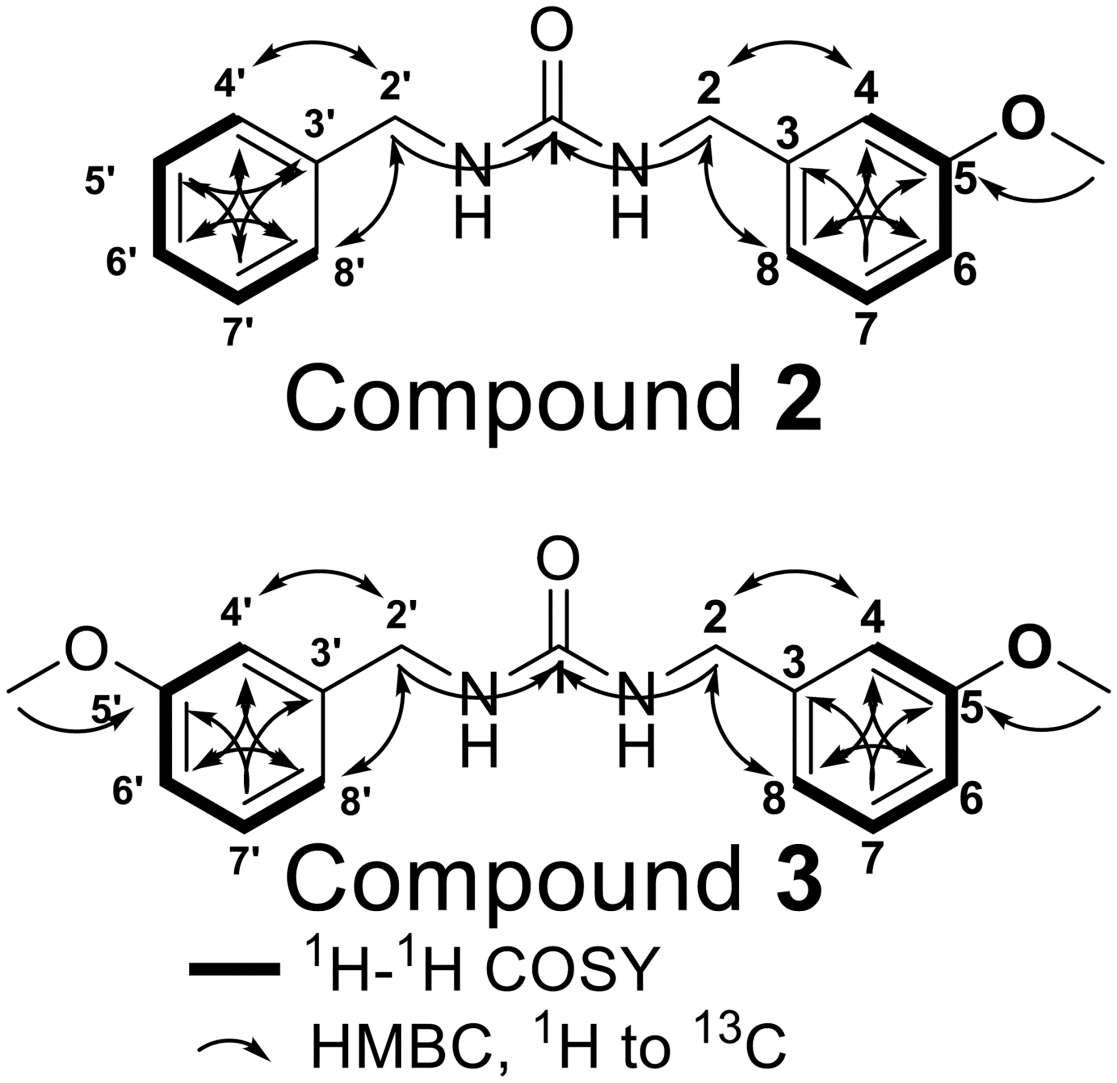
Key HMBC and ^1^H-^1^H COSY correlations of compound 2 and 3.

Maca root has been reported to contain *meta* methoxy substituted benzyl isothiocyanate in addition to non-substituted benzyl isothiocyanate [36,37], so we expected to find *meta* methoxy substituted benzylureas. To our knowledge, there are no reports of dibenzylurea derivatives from maca. Compound **1**, unsymmetric 1-benzyl-3-(3-methoxybenzyl) urea (compound **2**), and 1, 3-bis (3-methoxybenzyl) urea (compound **3**) were isolated from maca root, and the NMR data are shown in Figs 2 & 3 and S1–S3 Figs. All of the spectroscopic data are identical to the synthetic standards, supporting the structure of these urea compounds (S1–S3 Figs). Compound **2** has not been reported previously, while there is one report of compound **3** (called salvadourea) found in the plant *Salvadora persica* [19], a Brassicales species.

### Soluble epoxide hydrolase inhibitory potency and physical properties of dibenzylurea derivatives found in Brassicales

**Fig 4:**
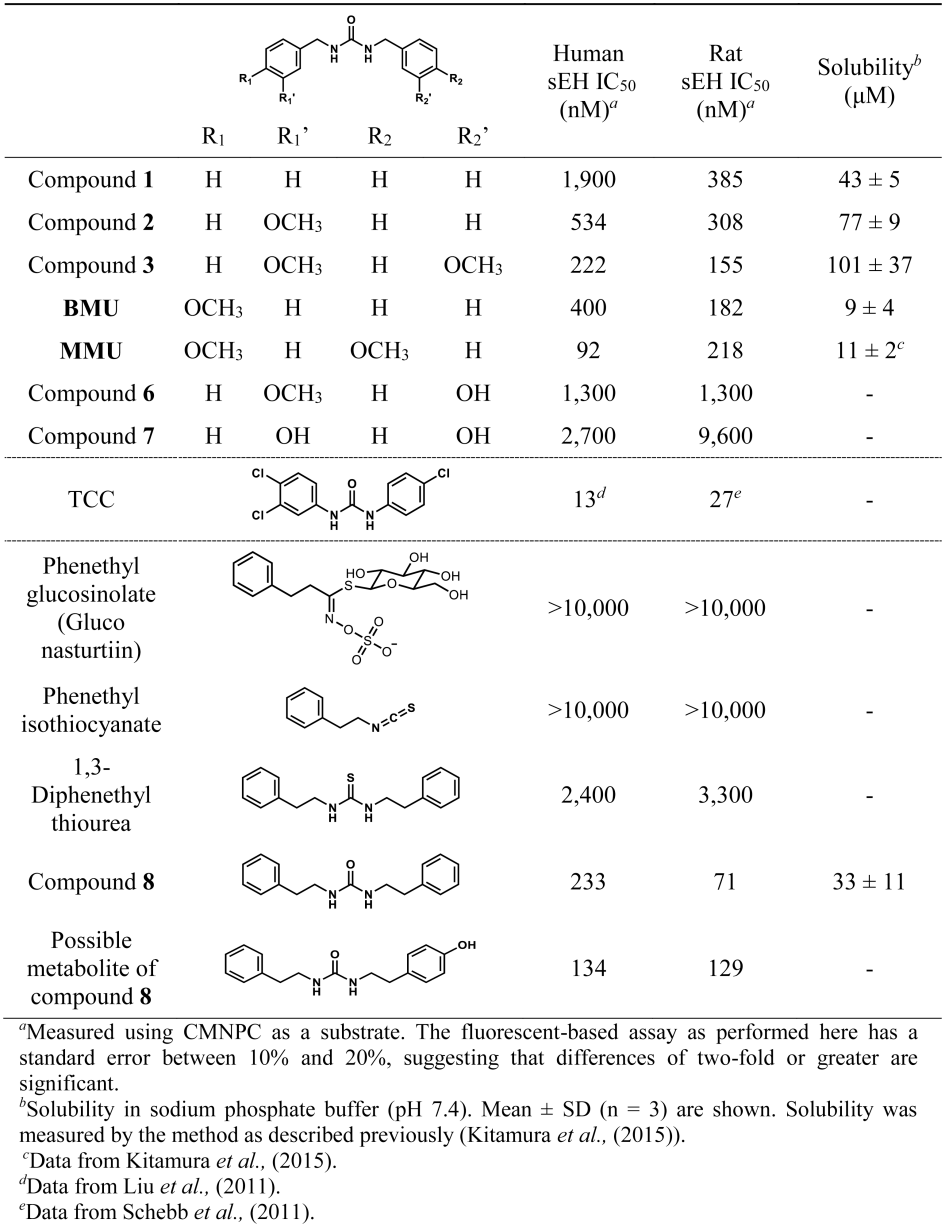
sEH inhibition potency of ureas found in Brassicales and related compounds.

The sEH inhibitory potency of benzylurea derivatives found in Brassicales was measured in order to understand their possible biological implications. As shown in Fig 4, compound **3** displayed inhibitory potency on human sEH with an IC_50_ value in the range of 200 nM, followed by compound **2** with approximately 2-fold lower potency. Compound **3** is approximately 2-fold less potent than the *para* methoxy substituted benzylurea derivatives (MMU) found in *P*. *brazzeana* [17,18], while the solubility of compound **3** is approximately 10-fold higher than MMU. These are among the most potent sEH inhibitors derived from natural sources, although they are still at least 100-fold less potent than highly potent synthetic inhibitors such as *trans*-4-(4-(3-(4-trifluoromethoxy-phenyl)-ureido)-cyclohexyloxy)-benzoic acid (*t*-AUCB) which has an IC_50_ below 1 nM on the human sEH [38].

### Brassicales-derived sEH inhibitor compound 3 effectively blocks inflammatory pain

**Fig 5:**
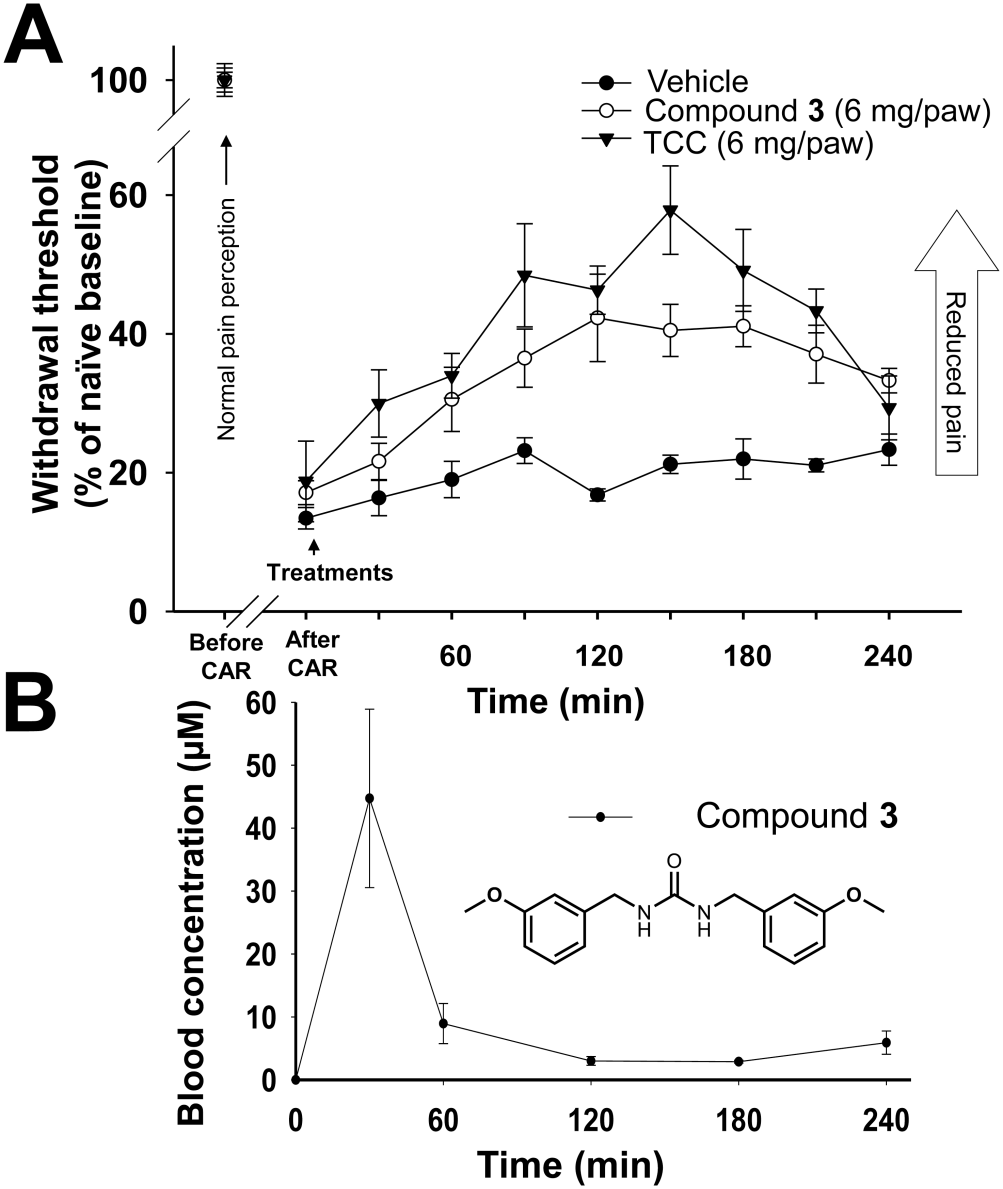
Topical treatment of compound 3 effectively reduces carrageenan-induced inflammatory pain in rat. (A) Carrageenan (CAR) induces a stable hyperalgesic response (enhanced pain perception) throughout the duration of the experiment. Treatment with compound **3** (○, 6 mg/paw) or synthetic sEH inhibitor (TCC) (▼, 6 mg/paw) significantly reduced pain levels (Kruskal-Wallis One Way ANOVA on Ranks, p ≤ 0.001, Tukey’s post hoc test (compound **3** vs vehicle, TCC vs vehicle, compound **3** vs TCC, p < 0.05). Mean ± SE (n = 6) of mechanical withdrawal threshold (% of naïve baseline) are shown. (B) Plasma concentration of compound **3** in the pain assay after dermal treatment of compound **3** (6 mg/paw). Concentration of demethoxy metabolite compounds **6** & **7** were below 200 nM. Blood samples were processed by the method described for pharmacokinetic analysis. Mean ± SE (n = 6) are shown.

Several studies have shown that sEH inhibitors effectively reduce inflammatory and neuropathic pain [4,5,7,12]. In order to evaluate the possible *in vivo* efficacy of plant urea derivatives, a rat inflammatory pain model was used and the analgesic effects of compound **3** were determined. Compound **3** was selected based on its inhibitory potency on sEH and its solubility. As shown in Fig 5, formulation in Vanicream and topical administration of either compound **3** (6 mg/paw) or the synthetic sEH inhibitor triclocarban (TCC, 6 mg/paw, positive control) significantly reduced carrageenan induced inflammatory pain (Kruskal-Wallis One Way ANOVA on Ranks, p ≤ 0.001). TCC is an antimicrobial agent widely used in personal care products, and is a potent synthetic sEH inhibitor with anti-inflammatory effects [39]. TCC possesses an IC_50_ of 27 nM on rat sEH [40], which is approximately 6-fold more potent than compound **3**. As expected, TCC showed slightly higher efficacy than compound **3** (Tukey’s post hoc test, compound **3** vs TCC, p < 0.05). The blood concentration of compound **3** was monitored during the assay (Fig 5B). At 30 min the concentration peaked, reaching a level higher than 40 μM, suggesting that compound **3** had high permeability through skin. Although the concentration rapidly decreased, even after 4 hours, compound **3** was detected at concentrations higher than 3 μM. It should be noted that local concentration of the compound in the inflamed paw could be higher than systemic concentration. A brief time lag between the compound concentration peak and the analgesic response was observed. This is consistent with earlier observations when an sEH inhibitors were administered topically [4].

### Brassicales-derived sEH inhibitor compound 3 is orally available

**Fig 6:**
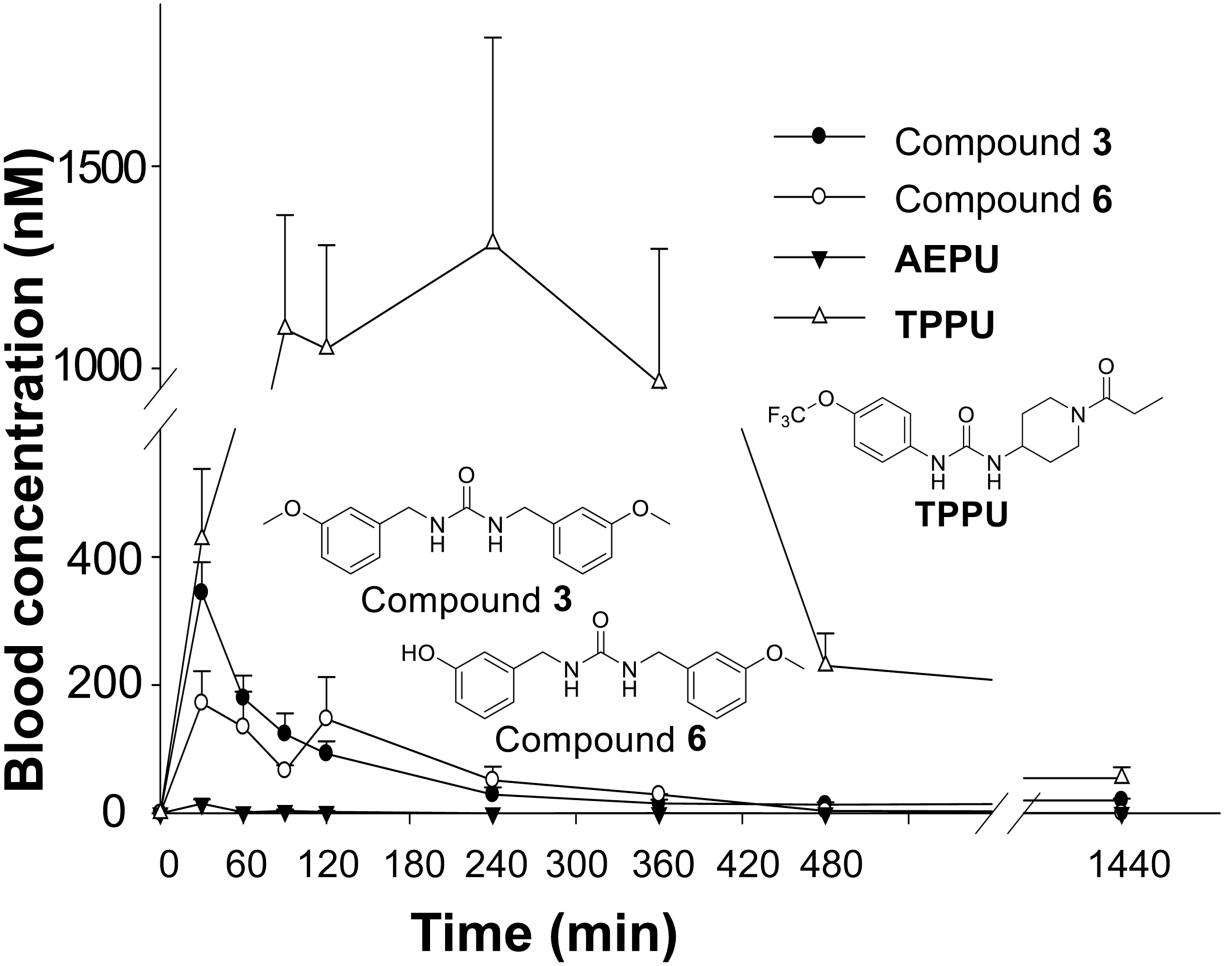
Compound 3 is orally bioavailable, and rapidly metabolized into demethoxy-metabolite compound 6. Male Swiss Webster mice were orally treated (by gavage) with 10 mg/kg of compound **3** and 1-(adamantan-1-yl)-3-(5-(2-(2-ethoxyethoxy) ethoxy) pentyl) urea (AEPU), and 0.3 mg/kg of 1-trifluoromethoxyphenyl-3-(1-propionylpiperidin-4-yl) urea (TPPU) formulated in PEG300 by cassette dosing (all three compounds given in one cassette). Compound **3**, a demethoxy-form metabolite of compound **3** (○, compound **6**), AEPU and TPPU were measured by LC-MS/MS. Bis-demethoxy compound (compound **7**) was not detected (LOQ < 9.8 nM in blood). Mean ± SE (n = 4) of blood concentrations are shown. Recently developed synthetic sEH inhibitor TPPU is much more bioavailable than many other compounds tested and is more metabolically stable. The data show compound **3** to be of sufficient oral availability and stability to be of possible pharmacological interest.

In order to determine pharmacokinetic properties, compound **3** was orally administered into mice. As shown in Fig 6, compound **3** was rapidly absorbed following oral administration. This result suggests that compound **3** could be absorbed when maca root powder is orally taken. It should be noted that maca contains various other ingredients which may interfere with absorption/distribution/metabolism, thus the pharmacokinetic profile of maca powder could be significantly different from our results. Two synthetic sEH inhibitors, 1-(adamantan-1-yl)-3-(5-(2-(2-ethoxyethoxy) ethoxy) pentyl) urea (AEPU) [27,41] and 1-trifluoromethoxyphenyl-3-(1-propionylpiperidin-4-yl) urea (TPPU) [12] were used as reference compounds. AEPU is an early-generation synthetic sEH inhibitor which showed efficacy on the development of atherosclerosis in a mouse model when delivered in drinking water [42]. As shown in Fig 6, the oral availability of compound **3** is higher than that of AEPU, while the more recently developed synthetic sEH inhibitor, TPPU is more stable and has much higher bioavailability than compound **3**. Compound **3** has relatively high solubility (Fig 4), and thus this compound may have better absorption compared to other urea derivatives found in Brassicales. The mono-demethoxy metabolite of compound **3** (compound **6**) was detected at similar concentrations to compound **3**. Based on its concentration and potency (human sEH IC_50_ = 1.3 μM, Fig 4), this metabolite could also contribute to the biological activity. Based on the area under the curve values of compound **3** and compound **6**, approximately 66% of compound **3** appears to be metabolized into compound **6**. On the other hand, the bis-demethoxy compound (compound **7**) was not detected (LOQ < 9.8 nM in blood). Phase II metabolism such as glucuronidation or sulfation may be the dominant pathways for the metabolism of compound **6**.

### Effect of incubation conditions on the occurrence of compound 1 from papaya seed

**Fig 7:**
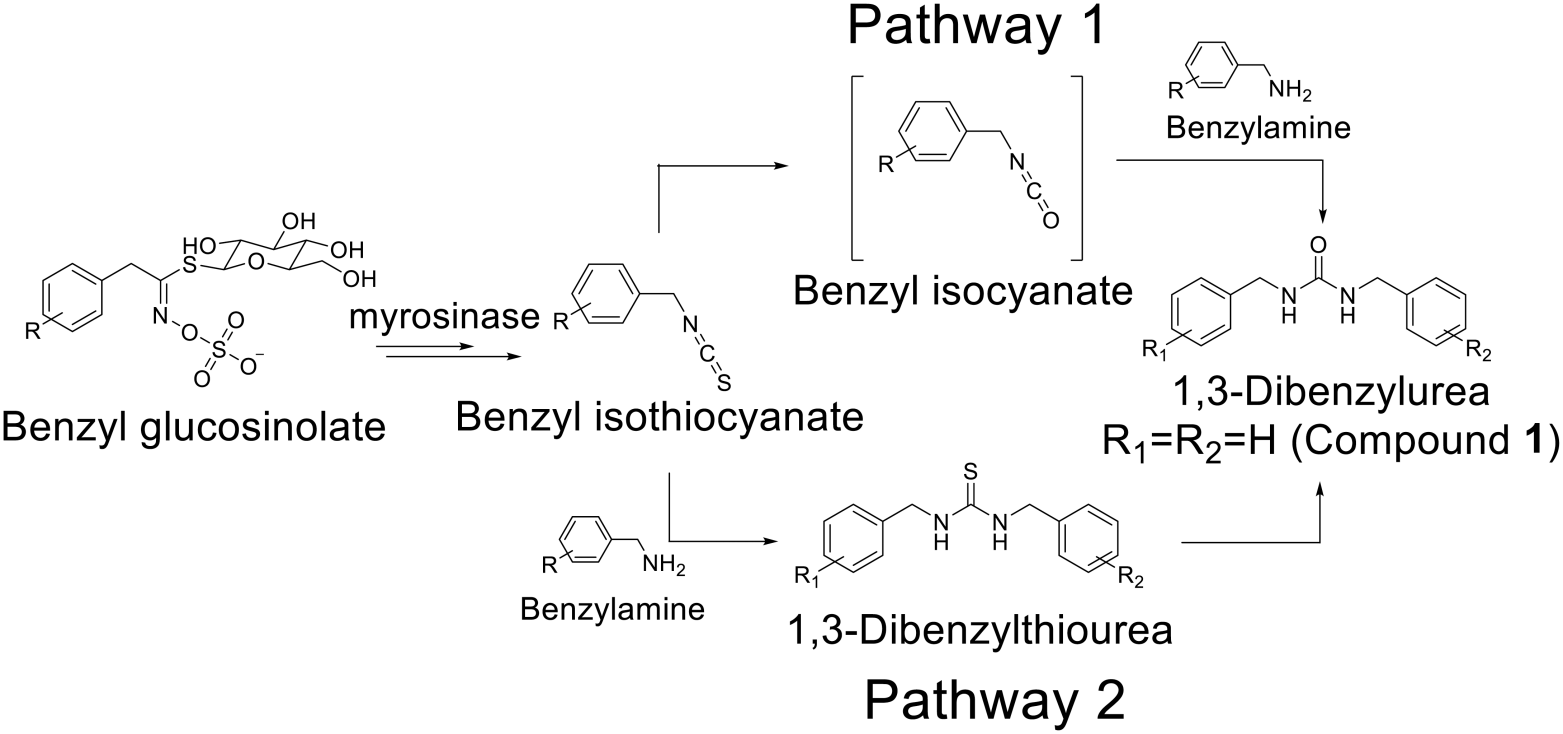
Proposed biosynthetic pathway of 1, 3-dibenzylurea derivatives.

Next, possible synthetic pathways for compound **1** was explored. We hypothesized the biosynthetic pathways of benzylurea derivatives in these plants as shown in Fig 7. Conversion of glucosinolates into isothiocyanates by myrosinase is a well-recognized metabolic pathway in Brassicales [43]. Conversion of benzyl isothiocyanate into benzyl isocyanate by human P450s has also been reported [44,45]. Although the oxidative enzyme that catalyzes this reaction in plants has not been identified, it is possible that plants also catalyze this reaction via a mechanism similar to that of human P450. Isocyanates can react with water to yield free amines or they can react directly with amines to form 1, 3-disubstituted ureas (pathway 1). Another possible pathway is the conversion of isothiocyanates into thioureas by reacting with benzylamines, which are then oxidized to 1, 3-dibenzylureas (pathway 2). Papaya seed was selected as a model system to study these pathways because of the ease of obtaining fresh samples and the relatively high concentration of benzyl isothiocyanate [46,47]. Moreover, it is incorporated into our diet [48] and is used as an active ingredient in an anthelmintic [35].

As shown in Table 2, compound **1** was not observed in samples without overnight incubation, demonstrating that compound **1** is produced during the incubation. This is consistent with our hypothesis that production of isothiocyanates during incubation is essential for the production of ureas. Treatment of samples with methanol, microwave heating, or liquid nitrogen prevented the production of compound **1**, strongly indicating the involvement of an enzymatic process. Compound **1** was not observed when the typical extraction procedure with liquid nitrogen for glucosinolates was used. This observation explains why these urea derivatives have been rarely detected in Brassicales.

**Table 2:** Effect of incubation conditions and trapping experiment on concentration of compounds 1 and 4 from papaya seed.

	Concentration of compound 1 (ng/g fresh seed weight)	Concentration of compound 4 (ng/g fresh seed weight)
Control (overnight incubation)	56 ± 11	<LOQ
Without overnight incubation	<LOQ	-
Methanol treatment	14 ± 5	-
Microwave heating (100°C 20 min in sealed tube)	7 ± 6	-
Liquid nitrogen	<LOQ	-
4-isopropylaniline (10 μM, 2 mL) spike	14 ± 0.5	975 ± 186
Compound **5** (10 μM, 2 mL) spike	43 ± 10	34 ± 9
Compound **5** (10 μM, 2 mL) spike without papaya	<LOQ	249 ± 18
1, 3-Dibenzylthiourea (10 μM, 2 mL) spike without papaya	561 ± 108	<LOQ

LOQ: 1.2 ng (compound **1**)/g and 2.6 ng (compound **4**)/g fresh seed weight. Mean ± SD values are shown.

### Analysis of isocyanate, isothiocyanate, and thiourea derivatives from papaya seed

Trapping experiments were performed in order to study the possible involvement of isocyanate, isothiocyanate, and thiourea in the biosynthesis of compound **1** [45]. These intermediates, especially the isocyanate, are chemically unstable and difficult to quantify. Both isocyanate and isothiocyanate can be trapped with 4-isopropylaniline, resulting in 1-benzyl-3-(4-isopropylphenyl) urea (compound **4**) and 1-benzyl-3-(4-isopropylphenyl) thiourea (compound **5**), respectively. An LC-MS/MS method was optimized in order to analyze these derivatives in addition to endogenous benzylurea and thiourea. Sufficient compound **4** was recovered with the same extraction method (Recovery: 107 ± 9%) used for the extraction of compound **1** from papaya samples, but the concentration and recovery percentage of 1, 3-dibenzylthiourea and compound **5** varied significantly, possibly because of the instability of the thiourea. Thus, only qualitative analysis was performed for these thiourea derivatives.

When 4-isopropylaniline was spiked into fresh papaya seeds, compound **4** was observed at high concentration and consistent with this, the level of compound **1** decreased slightly (Table 2). In addition, compound **5** was observed in these samples. Interestingly, compound **4** was also observed when compound **5** was spiked into papaya seed before incubation. This strongly suggests that biosynthetic pathway 2 is at least part of the synthetic pathway of urea derivatives. When compound **5** was incubated overnight in water at room temperature without papaya seed, surprisingly, we observed high levels of compound **4**. Similarly, 1, 3-dibenzylthiourea was converted into compound **1** under these conditions, suggesting that the oxidation of thiourea into urea could be a spontaneous non-enzymatic chemical reaction. Approximately 10% of 1, 3-dibenzylthiourea was converted into compound **1**. In addition, it should be noted that benzyl isocyanate was not observed in the GC-MS analysis of papaya seed (S7 Fig).

### Screening of plant library in the order Brassicales on sEH inhibition and occurrence of urea derivatives

A library of plants in the order Brassicales was constructed and screened for sEH inhibitory activity. We mainly focused on sprouts because of the following reasons; (1) relative high amount of glucosinolate and myrosinase activity was reported in Brassicales [49], (2) easy to obtain fresh samples, (3) sprouts of several Brassicales are used as food.

**Fig 8:**
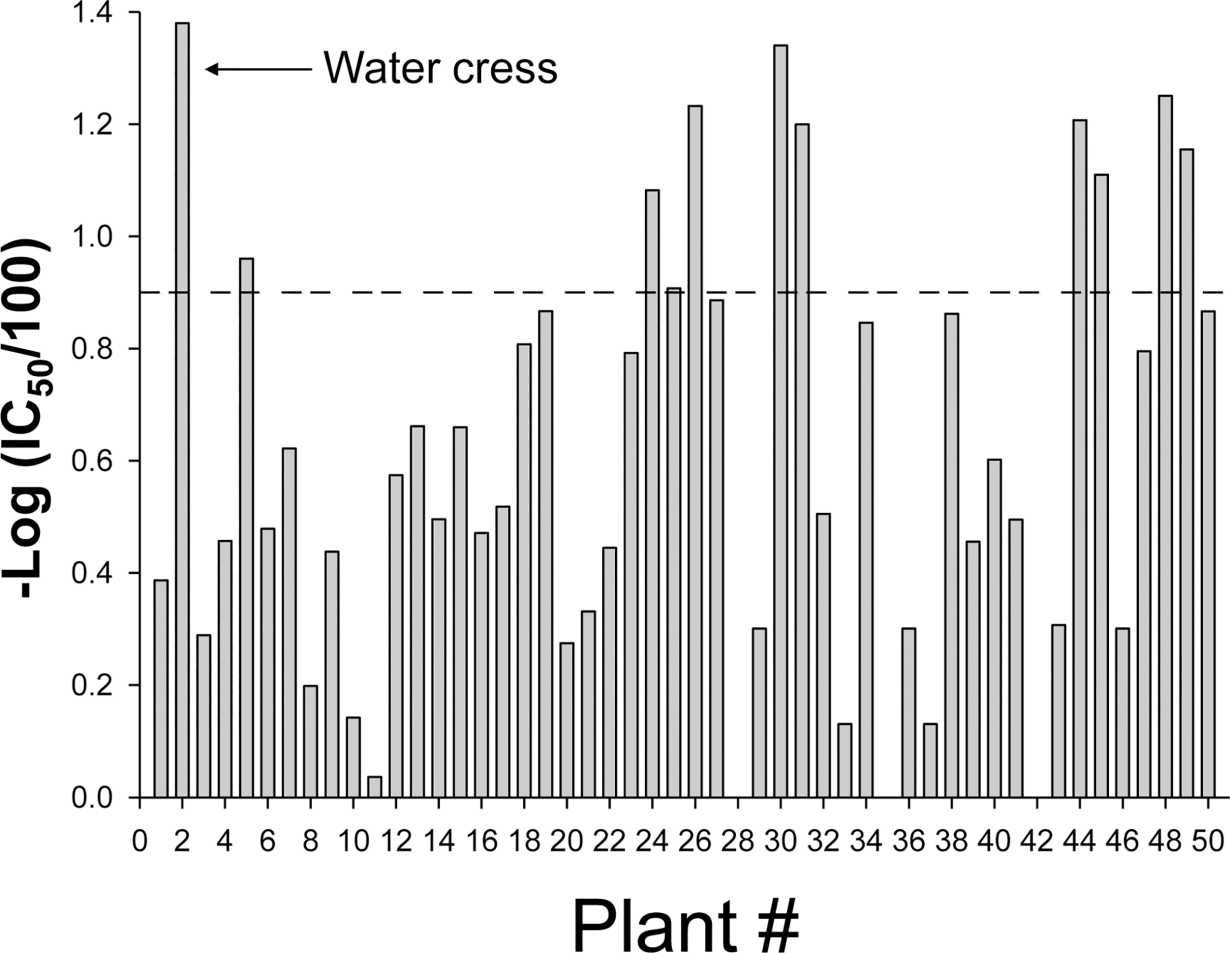
Screening results of plant library in the order Brassicales against human sEH inhibitory potency. IC_50_ was measured using cyano (2-methoxynaphthalen-6-yl) methyl *trans*-(3-phenyl-oxyran-2-yl) methyl carbonate (CMNPC) as a substrate. The fluorescent-based assay as performed here has a standard error between 10% and 20%, suggesting that differences of two-fold or greater are significant. Inhibitory potency was expressed as -log (IC_50_ (μg plant extract/mL)/100). Dotted line represents IC_50_ = 12.6 μg/mL. Extracts with IC_50_ above this value (below the dotted line on the graph) are considered not active. IC_50_ values and plant names are given in S3 Table.

As shown in Fig 8, screening using the fluorescent-based assay clearly prioritized the plant extracts with regard to sEH inhibition. Plants in the *Brassica* genus did not show potent activity except for Redcabbage (*Brassica oleracea*) even though the library contained 17 species in the genus *Brassica*. This tendency may be because this genus usually produces glucosinolates with short linear aliphatic chains such as sulforaphane [49–51]. According to the previous structure-activity relationship information, bulky group on at least one side of urea gives higher sEH inhibitory activity than short aliphatic chains [52].

The Brassicales library was then screened for the occurrence of urea derivatives using LC-MS/MS (S2 Table & Table 3). Compound **1** (1, 3-dibenzylurea) was found in the sprouts of garden nasturtium (*Tropaeolum majus*) and the root of Salvadora (*Salvadora persica*), in addition to garden cress and Moringa (*Moringa oleifera*). Garden nasturtium and Salvadora have been reported to produce benzyl glucosinolate [25,51,53]. Compound **8** (1, 3-diphenethyl urea) was detected in the sprout of land cress (*Barbarea verna*) at the highest concentration, although the extract showed only weak sEH inhibitory potency. The occurrence of compound **8** in land cress is consistent with previous reports that this plant is rich in phenethyl glucosinolate [51,54,55]. Given the high concentration of phenethyl glucosinolate in land cress, this plant may be ideal for further optimization of sEH inhibition. A trace amount of compound **8** was detected in sprouts of garden nasturtium, horse radish (*Armoracia rusticana*), and alyssum (*Lobularia maritima*), consistent with the previous reports of the occurrence of phenethyl glucosinolate or phenethyl isothiocyanate in these plants [56]. Methoxy benzyl urea was detected in the sprouts of Limnanthes (*Limnanthes douglasii*), maca, black and curled mustard (*Brassica nigra* and *juncea*), and the leaf of horse radish. Limnanthes has been reported to produce *meta* methoxy benzyl glucosinolate, thus it is likely that compound **3** (*meta* methoxy benzyl urea) was produced from this plant [51,55,57,58]. When comparing the occurrence of these urea derivatives with sEH inhibitory potency, water cress, Moringa, and maca, which produce urea derivatives, showed high inhibitory potency on sEH, while several other plants that produce urea derivatives showed limited inhibitory potency.

**Table 3:** Summary of the screening of Brassicales library for the occurrence of urea derivatives.

#	Plant	Part	Human sEH IC_50_ (μg extract/mL)	Compound 1 (nmol/g extract)	Compound 3 (or MMU, nmol/g extract)	Compound 8 (1,3-diphenetyl urea, nmol/g extract)
2	Water cress	sprout	1.4			129
3	Nasturtium	sprout	51.4	1524		43
4	Horse radish	root	34.9			34
6	Garden cress	sprout	33.2	1592		
7	Moringa	root	23.9	6576		
13	Land Cress	sprout	21.8			2820
26	Moringa	sprout	5.9	469		
28	Salvadora	root	100.0	53		
29	Limnanthes	sprout	50.0		7413	
30	Maca	root	4.5	18	10	
31	Maca	sprout	6.3		33	
32	Black mustard	sprout	31.3		25	
34	Alyssum	sprout	14.3			29
36	Water cress	seed	50.0			45
38	Curled mustard	sprout	13.7		13	
39	Horse radish	leaf	35.0		13	
50	Water cress	stem	13.6			21

Only detected urea derivatives are listed in the table. Note that the concentration is semi-quantitative, and matrix effects may interfere/enhance the ionization.

### Quantification of 1, 3-diphenethyl urea from water cress

**Fig 9:**
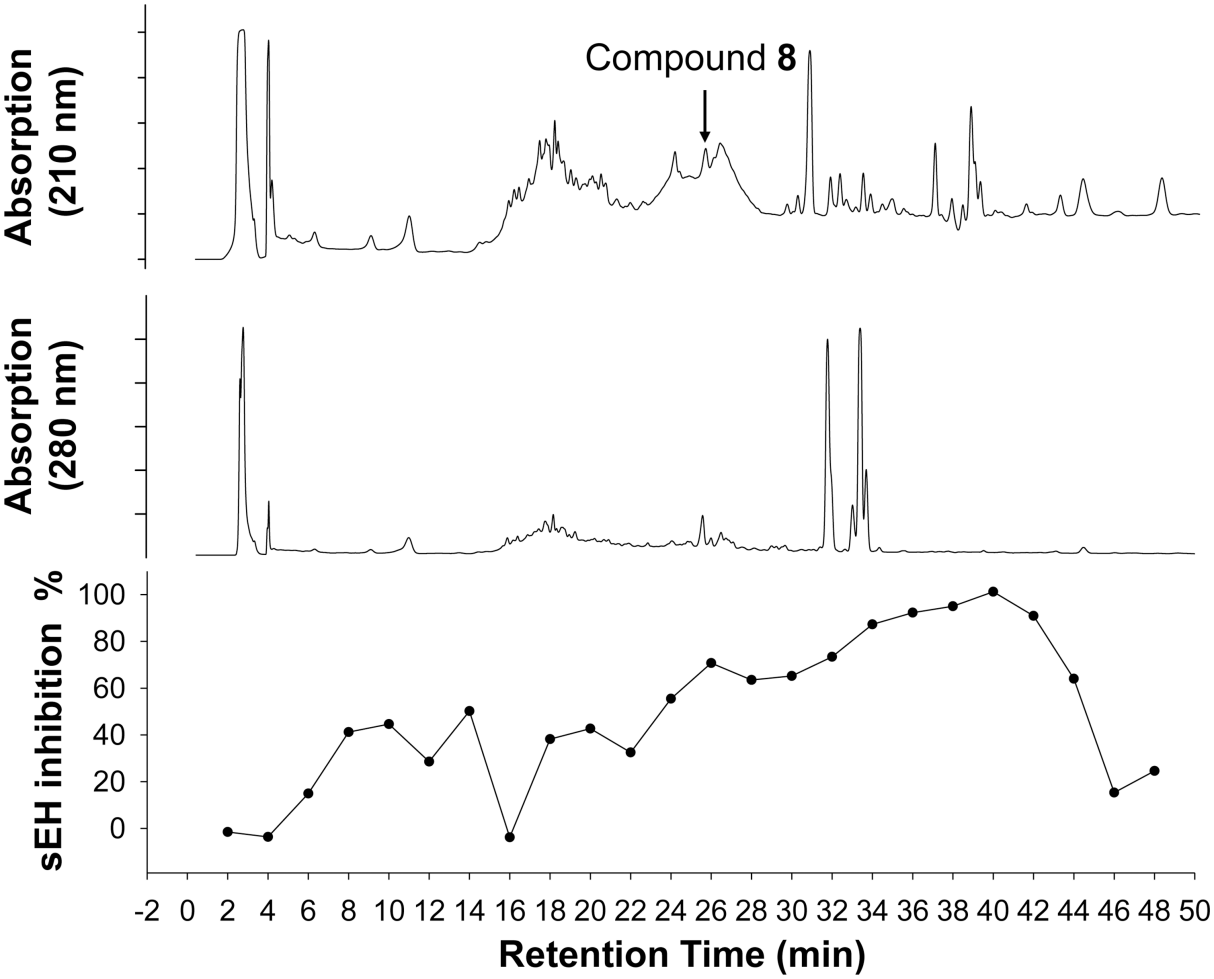
sEH inhibitory potency of HPLC fractions of water cress extract. Crude extract of water cress sprout (approximately mg) was injected into a reverse phase HPLC column (Waters SunFire Prep C18, 5 μm, 10x100 mm) and eluted with 10% acetonitrile in water with a flow rate of 2 mL/min for 10 min, followed by a linear gradient elution of acetonitrile 10% to 100% at a flow rate of 2 mL/min for 25 min, and eluted with 100% acetonitrile for 15 min at a flow rate of 2 mL/min. The relative intensity of the UV absorption at the wavelength of 210 and 280 nm are shown (top and middle). The retention time of the synthetic urea (compound **8**) is indicated by an arrow in the figure. Fractions were collected for every 4 mL of eluent. After the solvent was evaporated the residue was reconstituted in 50 μL DMSO. The inhibition percentage by each fractions (100 times dilution of reconstituted solution) was measured using the CMNPC assay with recombinant human sEH (bottom). The black circles (●) represent the inhibition percentage by each of the fractions. The crude extract (2 mg extract/mL) showed complete inhibition of sEH activity.

In order to validate the screening results, we focused on water cress for the following reasons; (1) high inhibitory potency on human sEH, (2) the ease of cultivation and availability of fresh vegetables, (3) reports of a high concentration of phenethyl glucosinolate [59]. Thus we focused on diphenethyl urea compound **8**, which may contribute to the observed sEH inhibitory potency of water cress extract. First, the sEH inhibitory potency of compound **8** and related compounds was measured (Fig 4). As expected from previous structure-activity relationships, compound **8** showed moderate sEH inhibitory potency, while phenethyl thiourea and isothiocyanate were not potent sEH inhibitors. These data suggest that conversion of thiourea into urea could increase the potency of extracts. Next, we looked for compound **8** in water cress. This compound was detected and quantified in various parts of the plant (Table 4). Spike and recovery experiments showed that our method sufficiently extracts compound **8** from the samples (Recovery: 80 ± 5%). Leaf samples showed the highest sEH inhibitory potency. As expected, leaf samples had the highest concentration of compound **8** when compared per extract weight. Interestingly, seed extracts did not show inhibitory potency, although they contain a moderate concentration of compound **8**, suggesting that components other than compound **8** may be responsible for the sEH inhibitory potency. To test this possibility, fraction collection experiments were performed (Fig 9, S2 Text and S5 Table). The fraction containing compound **8** showed only 5% of total inhibitory potency, while other fractions, especially lipophilic fractions showed high potency toward sEH. Identification and characterization of these components are subjects of further study. It should be noted that sprout extracts in these experiments showed much lower inhibitory potency compared to the samples in the initial screening. This discrepancy could be due to the different batches of the seed samples, or slight change of growth conditions.

**Table 4:** Concentration of compound 8 in various parts of water cress.

	Human soluble epoxide hydrolase IC_50_ (μg/g extract)	Concentration of compound 8 (μg/g extract)	Concentration of compound 8 (μg/g dry weight)	Concentration of compound 8 (μg/g fresh weight)
Leaf	7.0 ± 1.1	48 ± 15	2.7 ± 0.9	0.38 ± 0.09
Sprout	17 ± 2	3.2 ± 1.4	0.7 ± 0.3	0.11 ± 0.04
Stem	13.6 ± 1.0	11 ± 4	0.8 ± 0.3	0.07 ± 0.03
Seed	>50	12 ± 4	1.4 ± 0.2	1.4 ± 0.2

Mean ± SD values are shown.

Leaves of water cress are used as food, and several health benefits from their consumption have been reported [60]. Oral bioavailability as well as metabolism of compound **8** needs to be determined to relate the occurrence of compound **8**, sEH inhibition, and the health benefits of water cress. A possible hydroxylated metabolite of compound **8** showed slightly higher inhibitory potency on sEH than the parent, suggesting that this compound could also contribute to the biological activity *in vivo*.

A few papers have reported benzyl urea derivatives from plants, but their synthetic pathways or biological activities have not been studied extensively. Based on our data, it is likely that conversion of thiourea into urea (Fig 7, pathway 2) is at least part of the natural synthetic pathway of the urea derivatives reported in these earlier studies, given the processing method they reported. To our knowledge there are no reports of spontaneous oxidation of thiourea into urea without a special chemical reagent or catalyst. There are several synthetic methods to convert thiourea into urea such as using 1-butyltriphenylphosphonium dichromate, potassium monopersulfate [61], or bromine water within the pH range 2–4 [62]. Compared to these, our method is simple and mild (neutral pH at RT), and does not require any chemical reagent except for water and possibly oxygen from air. Determining the mechanism and substrate specificity of the oxidation reaction should help determine whether other disubstituted ureas occur in Brassicales. Moreover, optimization of this reaction should increase the yield of urea-based sEH inhibitors from Brassicales.

Because the conversion of thioureas into ureas is a spontaneous chemical reaction, we predict that the occurrence of urea derivatives is conserved among Brassicales that produce glucosinolate and isothiocyanate. Plants that have been reported to contain benzylurea derivatives have diverse genetic backgrounds and are widely spread among families in Brassicales. Determining the generality of the occurrence of urea derivatives in other Brassicales might lead to the discovery of more potent and metabolically stable urea-based sEH inhibitors from Brassicales.

In practice, urea derivatives may be generated during the preparation of traditional medicines or cooking process of vegetables from plants in the Brassicales. Soluble epoxide hydrolase inhibitors have been reported to be effective treatments in various animal disease models, including models of inflammatory and diabetic neuropathic pain [4,5,7,12], hypertension [63], cardiac hypertrophy [64], chronic obstructive pulmonary disease [65], and fibrosis [66]. Moreover, in combination with omega-3 fatty acid metabolite or cyclooxygenase-2 inhibition, sEH inhibitors synergistically inhibit angiogenesis thus effectively suppress tumor growth and metastasis [67,68]. Urea-based sEH inhibitors may contribute, at least in part, to the beneficial effects of Brassicales, in addition to the well-known Nrf2-Keap1 pathway modulated by isothiocyanates [49,69,70]. We believe that these urea derivatives are an important class of chemicals that occur in nature, or more importantly, occur in our daily life.

These urea derivatives have potential for nutraceutical or pharmaceutical application for the treatment of pain and other pathological conditions in which sEH inhibitors are effective. From the point of view of nutraceutical or clinical application for the treatment of pain, a topical route is convenient and may be advantageous considering the rapid metabolism and high permeability of the compound through skin.

In addition to sEH inhibitors, the 1, 3-disubstituted urea moiety is frequently used in medicinal chemistry and is reported to possess various biological activities [71–78]. For example, many of the kinase inhibitors, including the marketed anticancer drugs sorafenib and regorafenib, have 1, 3-disubstituted urea-based structure with antitumor activity [79]. Urea derivatives from Brassicales may interact with these target proteins.

## Conclusions

Urea derivatives were detected in multiple plants in the order Brassicales, and their synthetic pathways were studied, suggesting a thiourea as an intermediate. *Meta* methoxy substituted dibenzylurea derivatives that possess sEH inhibitory activity were isolated and characterized from maca root. The most potent compound (compound **3**) showed the expected analgesic efficacy on inflammatory pain and was orally available. Although urea derivatives in nature have not been studied extensively, this class of compounds could provide unique chemical diversity in the field of natural products and may have implications for human health.

## Supporting information

S1 Text DNA extraction and sequencing of ribosomal DNA partial sequences. (PDF)

S2 Text Methods for HPLC fraction collection and sEH inhibition by the fractions. (PDF)

S1 Table Optimum mass transition conditions and key fragmentation of ureas and thioureas. (PDF)

S2 Table List of urea/thiourea derivatives, mass transition conditions, and key fragmentation for MS screening of Brassicales plant library. (PDF)

S3 Table HPLC solvent gradients for the analysis of urea compounds. (PDF)

S4 Table Information of Brassicales plant library. (PDF)

S5 Table Relative potency of reverse phase-HPLC fractions. (PDF)

S1 Fig NMR spectra of compound 1 (synthetic standard & sample isolated from maca). (PDF)

S2 Fig NMR spectra of compound 2 (synthetic standard & sample isolated from maca). (PDF)

S3 Fig NMR spectra of compound 3 (synthetic standard & sample isolated from maca. (PDF)

S4 Fig HRESIMS spectra of compound 1 isolated from maca. (PDF)

S5 Fig HRESIMS spectra of compound 2 isolated from maca. (PDF)

S6 Fig HRESIMS spectra of compound 3 isolated from maca. (PDF)

S7 Fig GC-MS analysis of benzyl isocyanate and benzyl isothiocyanate. (PDF)

S8 Fig Intraplantar administration of compound 3 effectively reduces carrageenan-induced inflammatory pain in rat. (PDF)
