# Supplementary material for: Occurrence of urea-based soluble epoxide hydrolase inhibitors from the plants in the order Brassicales
Source: PLoS One. 2017 May 4;12(5):e0176571. doi: 10.1371/journal.pone.0176571 (PMC5417501; doi:10.1371/journal.pone.0176571)
Supplement: S1 Text — (PDF) [file pone.0176571.s001.pdf]

S1 Text. DNA extraction and sequencing of ribosomal DNA partial sequences.

Total DNA in the maca root powder was purified by phenol-chloroform extraction followed by ethanol precipitation, and suspended in 10 mM Tris-HCl (pH 8.0)[1]. The partial sequences of 18S and 5.8S ribosomal DNA were amplified by PCR. The sequences of the PCR primers are as follows: 18S ribosomal DNA, forward primer-1 (5'-GATCAGATACCGTCCTAGTCTCAACC-3') and reverse primer-1 (5'-CCTTGTTACGACTTCTCCTTCCTCTA-3'); and 5.8S ribosomal DNA, forward primer-1 (5'- GAAAAGTGTCAAGGAACATGCAAC-3') and reverse primer-1 (5'-CAATATGAAGAGGCTTTAGATCACCA-3'). PCR amplification was performed with these primers using GoTaq Green Master Mix (Promega, Madison, WI, USA) as follows: 95 ºC, 2 min; 30 cycles of 95 ºC, 30 sec; 62 ºC, 30 sec; and 72 ºC, 45 sec; followed by 72 ºC, 5 min. This PCR generated a 0.8 kbp (18S) or 0.4 kbp (5.8S)-long amplicon. Nested PCR amplification was performed with the PCR product. The PCR primers are as follows: 18S ribosomal DNA, forward primer-2 (5'- GGTCCAGACATAGTAAGGATTGACAGAC-3') and reverse primer-2 (5'- GATAAGGTTTAGTGGACTTCTCGCG-3'); 5.8S ribosomal DNA, forward primer-2 (5'- CTGTGCTGCGATCTAAAGTCTAAAACGAC-3') and reverse primer-2 (5'- AATTTTGTGAGGGGAACGACGATTTG-3'). This PCR generated a 0.5 kbp (18S) or 0.2 kbp (5.8S)-long amplicon that was column-purified using a QIAquick Gel Extraction Kit (Qiagen, Valencia, CA, USA) following the manufacturer's protocol. The sequences of PCR products were determined by the UC Davis College of Biological Sciences Sequencing Facility. Sequence alignments of PCR products from maca dried root sample vs. sequences in NCBI database are shown below.

18s rDNA

Query: NCBI accession number AY544990 Lepidium meyenii 18S ribosomal RNA gene, partial sequence

Subject 1: Sequence of 18s rDNA from root of maca

Score	Expect	Identities	Gaps	Strand
863 bits(467)	0.0	482/488(99%)	6/488(1%)	Plus/Plus

```
Query  1225  ATTGACAGACTGAGAGCTCTTTCTTGATTCTATGGGTGGTGGTGCATGG-CGTTCTTAGT  1283
             ||||||||||||||||||||||||||||||||||||||||||||||||| ||||||||||
Sbjct  1     ATTGACAGACTGAGAGCTCTTTCTTGATTCTATGGGTGGTGGTGCATGGCCGTTCTTAGT  60


Query  1284  TGGTGGAGCGATTTGTCTGGTTAATTCCGTTAACGAACGAGACCTCAGCCTGCTAACTAG  1343
             ||||||||||||||||||||||||||||||||||||||||||||||||||||||||||||
Sbjct  61    TGGTGGAGCGATTTGTCTGGTTAATTCCGTTAACGAACGAGACCTCAGCCTGCTAACTAG  120
```

```
Query  1344  CTA-GTGGAGGCATCCCTTCACGGCCGGCTTCTTAGAGGGACTATGGCCGTTTAGGCCAA  1402
             ||| ||||||||||||||||||||||||||||||||||||||||||||||||||||||||
Sbjct  121   CTACGTGGAGGCATCCCTTCACGGCCGGCTTCTTAGAGGGACTATGGCCGTTTAGGCCAA  180

Query  1403  GGAAGTTTGAGGCAAT-ACAGGTCTGTGATGCCCTTAGATGTTCTGGGCCGCACGCGCGC  1461
             |||||||||||||||| |||||||||||||||||||||||||||||||||||||||||||
Sbjct  181   GGAAGTTTGAGGCAATAACAGGTCTGTGATGCCCTTAGATGTTCTGGGCCGCACGCGCGC  240

Query  1462  TACACTGATGTATTCAACGAGTTCACACC-TGGCCGACAGGCCCGGGTAATCTTTGAAAT  1520
             ||||||||||||||||||||||||||||| ||||||||||||||||||||||||||||||
Sbjct  241   TACACTGATGTATTCAACGAGTTCACACCTTGGCCGACAGGCCCGGGTAATCTTTGAAAT  300

Query  1521  TTCATCGTGATGGGGATAGATCATTGCAATTGTTGGTCTTC-ACGAGGAATTCCTAGTAA  1579
             ||||||||||||||||||||||||||||||||||||||||| ||||||||||||||||||
Sbjct  301   TTCATCGTGATGGGGATAGATCATTGCAATTGTTGGTCTTCAACGAGGAATTCCTAGTAA  360

Query  1580  GCGCGAGTCATCAGCTCGCGTTGACTACGTCCCTGCCCTTTGTACACACCGCCCG-CGCT  1638
             ||||||||||||||||||||||||||||||||||||||||||||||||||||||| ||||
Sbjct  361   GCGCGAGTCATCAGCTCGCGTTGACTACGTCCCTGCCCTTTGTACACACCGCCCGTCGCT  420

Query  1639  CCTACCGATTGAATGATCCGGTGAAGTGTTCGGATCGCGGCGACGTGGGTGGTTCGCCGC  1698
             ||||||||||||||||||||||||||||||||||||||||||||||||||||||||||||
Sbjct  421   CCTACCGATTGAATGATCCGGTGAAGTGTTCGGATCGCGGCGACGTGGGTGGTTCGCCGC  480

Query  1699  CTGCGACG  1706
             ||||||||
Sbjct  481   CTGCGACG  488
```

5.8s rDNA

Query: NCBI accession number JX908826 Lepidium meyenii internal transcribed spacer 1, 5.8S ribosomal RNA gene, and internal transcribed spacer 2, complete sequence

Subject 1: Sequence of 5.8S rDNA from root of maca

Score	Expect	Identities	Gaps	Strand
355 bits(192)	9e-103	192/192(100%)	0/192(0%)	Plus/Plus

```
Query  255  CGATCTAAAGTCTAAAACGACTCTCGGCAACGGATATCTCGGCTCTCGCATCGATGAAGA  314
            ||||||||||||||||||||||||||||||||||||||||||||||||||||||||||||
```

```
Sbjct  1    CGATCTAAAGTCTAAAACGACTCTCGGCAACGGATATCTCGGCTCTCGCATCGATGAAGA  60

Query  315  ACGTAGCGAAATGCGATACTTGGTGTGAATTGCAGAATCCCGTGAACCATCGAGTCTTTG  374
            ||||||||||||||||||||||||||||||||||||||||||||||||||||||||||||
Sbjct  61   ACGTAGCGAAATGCGATACTTGGTGTGAATTGCAGAATCCCGTGAACCATCGAGTCTTTG  120

Query  375  AACGCAAGTTGCGCCCCAAGCCTTCTGGCCGAGGGCACGTCTGCCTGGGCGTCACAAATC  434
            ||||||||||||||||||||||||||||||||||||||||||||||||||||||||||||
Sbjct  121  AACGCAAGTTGCGCCCCAAGCCTTCTGGCCGAGGGCACGTCTGCCTGGGCGTCACAAATC  180

Query  435  GTCGTTCCCCTC  446
            ||||||||||||
Sbjct  181  GTCGTTCCCCTC  192
```

References

1. Sambrock, J.; Fritsch, E. F.; Maniatis, T., Molecular Cloning, A Laboratory Manual (Second Edition), Volumes 1, 2 and 3. Cold Spring Harbor 1989. Cold Spring Harbor Laboratory Press. ISBN: 0-87969-309-6. **1989**.
