# Supplementary material for: Occurrence of urea-based soluble epoxide hydrolase inhibitors from the plants in the order Brassicales
Source: PLoS One. 2017 May 4;12(5):e0176571. doi: 10.1371/journal.pone.0176571 (PMC5417501; doi:10.1371/journal.pone.0176571)
Supplement: S2 Text — (PDF) [file pone.0176571.s002.pdf]

S2 Text. Methods for HPLC fraction collection and sEH inhibition by the fractions

HPLC fractionation was performed on an Agilent 1200 Series HPLC with a G1322A degasser, a G1311A Quatpump, and a G1315D Agilent detector. The crude sprout extract of water cress was prepared by the method described in the Materials and Methods. 19 mg of the crude extract was dissolved into 190 μL of MeOH, and 100 μL of the soluble fraction (approximately equivalent to 10 mg of the extract) was injected into a reverse phase HPLC column (Waters SunFire Prep C18 5μm, 10x100 mm). Analytes were eluted with 10% acetonitrile in water with a flow rate of 2 mL/min for 10 min, followed by a linear gradient elution of acetonitrile 10% to 100% at a flow rate of 2 mL/min for 25 min, and eluted with 100% acetonitrile for 15 min at a flow rate of 2 ml/min.

The UV absorption at 210 nm and 280 nm was monitored. After sample injection, 4 ml-fractions were collected. The solvent was evaporated and the residue was reconstituted in 50 μL DMSO. In parallel to these fractions, crude extract was dissolved into 4 mL of acetonitrile:water (1:1), evaporated, and reconstituted in 50 μL DMSO to compare the potency with fractions.

The inhibitory potency on human sEH was measured using the CMNPC assay as described in the Materials and Methods. Firstly, the inhibition percentage by each fraction (100 times dilution) was measured. Then the inhibitory potency was measured for the fractions showing higher than 50% inhibition and the IC_{50} was determined. The potency of each of the fractions is presented as a relative potency The reverse phase HPLC fraction collection resulted in the inhibitory recovery of 68% of crude extract.
