# Supplementary material for: Occurrence of urea-based soluble epoxide hydrolase inhibitors from the plants in the order Brassicales
Source: PLoS One. 2017 May 4;12(5):e0176571. doi: 10.1371/journal.pone.0176571 (PMC5417501; doi:10.1371/journal.pone.0176571)
Supplement: S1 Table — (PDF) [file pone.0176571.s003.pdf]

S1 Table. Optimum mass transition conditions and key fragmentation of ureas and thioureas

	Ionization mode	Transition			Cone voltage (V)	Collision voltage (V)
Compound **1**	+	241.2	->	91	30	20
1,3-dibenzylthiourea	+	257.1	->	91	10	20
Compound **4**	+	269.2	->	91	36	29
Compound **5**	+	285.1	->	91	50	20
1, 3-diphenylurea	+	213.1	->	94	35	16
Compound **3**	+	301.2	->	121	35	20
Compound **6**	+	278	->	121	40	30
Compound **7**	+	273	->	107	40	20
AEPU	+	397	->	220	30	25
TPPU	+	360.3	->	183	35	16
