# Supplementary material for: Occurrence of urea-based soluble epoxide hydrolase inhibitors from the plants in the order Brassicales
Source: PLoS One. 2017 May 4;12(5):e0176571. doi: 10.1371/journal.pone.0176571 (PMC5417501; doi:10.1371/journal.pone.0176571)
Supplement: S2 Table — (PDF) [file pone.0176571.s004.pdf]

**S2 Table.** List of urea/thiourea derivatives, mass transition conditions, and key fragmentation for MS screening of Brassicales plant library

Class 1. Included in the standard, and semi-quantified

| | hsEH $IC_{50}$ | | | | | Cone voltage (V) | Collision voltage (V) |
|---|---|---|---|---|---|---|---|
| Compound **1** 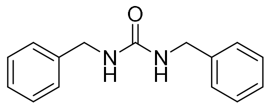 | 1900 | + | 241.2 | -> | 91 | 30 | 20 |
| **MMU** (&Compound **3** (meta)) 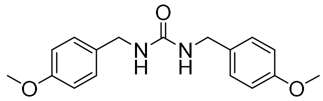 | 92 | + | 301.2 | -> | 121 | 35 | 20 |
| **BMU** (&Compound **2** (meta)) 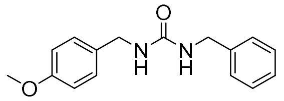 | 400 | + | 271.3 | -> | 121 | 33 | 23 |
| Compound **6** 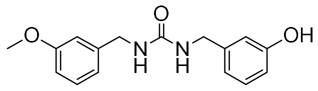 | 1300 | + | 278 | -> | 121 | 40 | 30 |
| Compound **7** 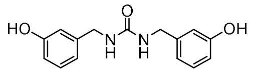 | 2700 | + | 273 | -> | 107 | 40 | 20 |
| Compound **8** 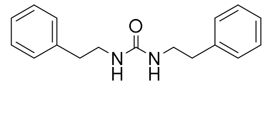 | 233 | + | 269 | -> | 105 | 35 | 30 |
| Compound **9** 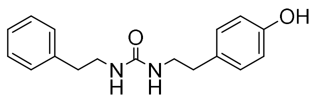 | 134 | + | 285 | | 121 | 35 | 25 |

| | | | | | | | |
|---|---|---|---|---|---|---|---|
| Compound **10** 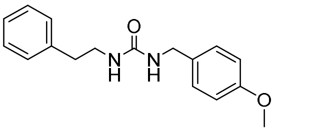 | 71.6 | + | 285 | -> | 121 | 35 | 25 |
| 1, 3-diphenylurea 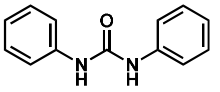 | - | + | 213.1 | -> | 94 | 35 | 16 |

Class 2: MS transition included, but not included in the standard (Thioureas)

| | | | | | | | |
|---|---|---|---|---|---|---|---|
| 1,3-diphenetylthiourea 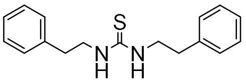 | - | + | 285 | -> | 105 | 30 | 25 |
| 1,3-dibenzylthiourea 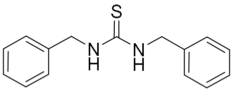 | - | + | 257 | -> | 91 | 35 | 25 |

Class 3: Without standard, predicted m/z

| | | | | | | | |
|---|---|---|---|---|---|---|---|
| 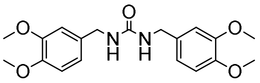 | - | + | 361 | -> | 151 | 35 | 40 |
| 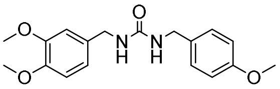 | - | + | 331 | -> | 167 | 35 | 20 |
| 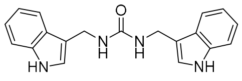 | - | + | 319 | -> | 130 | 35 | 40 |
| 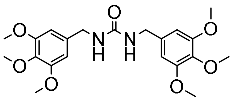 | - | + | 421 | -> | 181 | 35 | 40 |

| | | | | | | | |
|---|---|---|---|---|---|---|---|
| 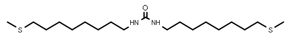 | - | + | 377 | -> | 159 | 35 | 40 |
| 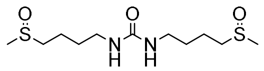 | - | + | 297 | -> | 119 | 35 | 40 |
| 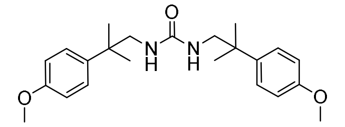 | - | + | 385 | -> | 163 | 35 | 40 |
