# Supplementary material for: Occurrence of urea-based soluble epoxide hydrolase inhibitors from the plants in the order Brassicales
Source: PLoS One. 2017 May 4;12(5):e0176571. doi: 10.1371/journal.pone.0176571 (PMC5417501; doi:10.1371/journal.pone.0176571)
Supplement: S3 Table — (PDF) [file pone.0176571.s005.pdf]

S3 Table. HPLC solvent gradients for the analysis of urea compounds

HPLC solvent gradient for the analysis of target ureas in plant samples

Time[a]	Aqueous phase[b]	Organic phase[c]
0.00	50	50
4.50	30	70
4.51	0	100
7.00	0	100

[a]0.25 mL/min flow rate, [b]Milli-Q water 99.9, acetic acid 0.1, volume %, [c]Acetonitrile 99.9, acetic acid 0.1, volume %.

HPLC solvent gradient for the analysis of target ureas in pharmacokinetic experiments

Time[a]	Aqueous phase[b]	Organic phase[c]
0.00	75	25
12.00	40	60
12.01	0	100
15.00	0	100

[a]0.25 mL/min flow rate, [b]Milli-Q water 99.9, acetic acid 0.1, volume %, [c]Acetonitrile 99.9, acetic acid 0.1, volume %.

HPLC solvent gradient for the purity determination

Time	Aqueous phase[a]	Organic phase[b]	Flow rate (mL/min)
0.00	90	10	0.3
15.00	0	100	0.6
25.00	0	100	0.6
25.01	90	10	0.3
35.00	90	10	0.3

[a]Milli-Q water 99.9, Formic acid 0.1, volume %, [b]Acetonitrile 99.9, Formic acid 0.1, volume %.

Purity determination of synthetic compounds was performed on an Agilent 1200 Series HPLC with a G1322A degasser, a G1311A Quatpump, and a G1315D Agilent detector. The Varian Pursuit5 C18 RP HPLC column (150 mm × 2.1 mm, particle size 5 µm) was used. The UV absorption between 190 nm and 400 nm was monitored, and the purity was determined by the peak area at 254 nm.

HPLC solvent gradient for the MS screening of urea derivatives on Brassicales plant library

Time[a]	Aqueous phase[b]	Organic phase[c]
0.00	90	10
6.00	90	10
6.01	30	70
10.50	0	100
15.00	0	100

[a]0.25 mL/min flow rate, [b]Milli-Q water 99.9, acetic acid 0.1, volume %, [c]Acetonitrile 99.9, acetic acid 0.1, volume %.
