# Supplementary material for: Occurrence of urea-based soluble epoxide hydrolase inhibitors from the plants in the order Brassicales
Source: PLoS One. 2017 May 4;12(5):e0176571. doi: 10.1371/journal.pone.0176571 (PMC5417501; doi:10.1371/journal.pone.0176571)
Supplement: S4 Table — (PDF) [file pone.0176571.s006.pdf]

S4 Table: Information of Brassicales library

#	Plant name	Part	Family	Scientific name	Product detail	Vendor name	HsEH IC_{50} (μg/mL)
**1**	Broccoli	sprout	Brassicaceae	*Brassica oleracea*	Broccoli Raab Certified Organic Heirloom Seeds 350 Seeds	Botanical Interests	41.0
**2**	Water cress	sprout	Brassicaceae	*Nasturtium officinale*	Organic Watercress Seeds - 200 mg - Nasturtium officinale	Botanical Interests	8.4
**3**	Nasturtium	sprout	Tropaeolaceae	*Tropaeolum majus*	Tall Single Blend Nasturtium Seeds - 3g (Botanical Interests)	Botanical Interests	51.4
**4**	Horse radish	root	Brassicaceae	*Armoracia rusticana*	Horseradish Roots Natural Organic Ready to Plant 1 Pound	Growers solution	34.9
**5**	Arabidopsis	sprout	Brassicaceae	*Arabidopsis thaliana* Ler	*Arabidopsis thaliana* Ler	LEHLE SEEDS	11.0
**6**	Garden cress	sprout	Brassicaceae	*Lepidium sativum*	Wrinkled Crinkled Crumpled Cress Seeds - 750 mg - Organic	Botanical Interests	33.2
**7**	Moringa	root	Moringaceae	*Moringa oleifera*	Moringa Tree Root Organic	Healing Moringa Tree	23.9
**8**	Daikon	sprout	Brassicaceae	*Raphanus sativus*	Radish Daikon (Miyashige White, Certified Organic)	Botanical Interests	63.3
**9**	Cabbage	sprout	Brassicaceae	*Brassica oleracea*	Copenhagen Market Cabbage Seeds - 1.5 grams	Botanical Interests	36.5
**10**	Collards	sprout	Brassicaceae	*Brassica oleracea*	Georgia Southern Collards Seeds - 6 grams - Heirloom	Botanical Interests	72.1
**11**	Kale	sprout	Brassicaceae	*Brassica oleracea*	Kale italian lacinato Nero toscana - 1 gram	Botanical Interests	91.9
**12**	mizuna	sprout	Brassicaceae	*Brassica rapa nipposinica*	Mustard Greens Mizuna	Botanical Interests	26.7
**13**	Land Cress	sprout	Brassicaceae	*Barbarea verna*	Upland Cress Heirloom Seeds - 2 grams	Botanical Interests	21.8
**14**	Radish	sprout	Brassicaceae	*Raphanus sativus*	Cherry Belle Radish Seeds - 6 grams	Botanical Interests	31.9
**15**	Arugula	sprout	Brassicaceae	*Eruca sativa*	Arugula Rocket Salad (Certified Organic)	Botanical Interests	21.9
**16**	Turnip	sprout	Brassicaceae	*Brassica rapa*	Turnip Purple Top White Globe	Botanical Interests	33.8
**17**	Bok choy	sprout	Brassicaceae	*Brassica rapa*	Toy Choy Bok Choy Seeds - 200 mg	Botanical Interests	30.3

**18**	Mustard spinach	sprout	Brassicaceae	*Brassica rapa*	Mustard spinach Tendergreen	Botanical Interests	15.6
**19**	spicy green	sprout	Brassicaceae	*Brassica juncea* (hybrid)	Mustard "Spicy Green" Seeds	Botanical Interests	13.6
**20**	chinese kale	sprout	Brassicaceae	*Brassca Oleracea var.alboglabra*	Chinese kale (Kailaan, white flowered) Seed	Botanical Interests	53.1
**21**	Brussels Sprout	sprout	Brassicaceae	*Brassica oleracea*	Long Island Improved Brussels Sprouts Heirloom Seeds	Botanical Interests	46.6
**22**	China rose radish	sprout	Brassicaceae	*Raphanus sativus*	China Rose Radish Sprouts Seeds - 30 grams	Botanical Interests	35.9
**23**	Dames Rocket	sprout	Brassicaceae	*Hesperis matronalis*	1500 Dames Rocket Seeds/ Free Shipping	Carolina Seeds and Exotics	16.1
**24**	Yellow Mustard	sprout	Brassicaceae	*Sinapis alba*	Outsidepride Yellow Mustard - 5000 Seeds	Outsidepride: Herb Seed	8.3
**25**	Red cabbage	sprout	Brassicaceae	*Brassica oleracea*	Cabbage Red Acre Seed	Botanical Interests	12.4
**26**	Moringa	sprout	Moringaceae	*Moringa oreifera*	Organic Moringa Seeds (100). USDA Certified Organic. Premium Grade High Germination Rate PKM1 Herbs Seeds.	Organic Veda - AGF	5.9
**27**	Rutabaga	sprout	Brassicaceae	*Brassica napobrassica*	Seeds Rutabaga Prymorska-Maritime Organic Russian Heirloom Vegetable Seed	DC Earth	13.0
**28**	Salvadora	root	Salvadoraceae	*Salvadora persica*	Sewak Al-Falah 5 pack	Sewak Al-Faleh	100.0
**29**	limnanthes	sprout	Limnamthaceae	*Limnanthes Douglasii*	Outsidepride Limnanthes Douglasii - 1000 Seeds	Outsidepride: Ground Cover Seed	50.0
**31**	maca	sprout	Brassicaceae	*Lepidium Meyenii*	100 Maca Root Seeds True Lepidium Meyenii - Natural Libedo Enhancer	ABCs of Gardening	6.3
**32**	black mustard	sprout	Brassicaceae	*Brassica nigra*	Hoosier Hill Farm Mustard Seeds, Black (1.5 pounds), 22 oz.	Hoosier Hill Farm	31.3
**33**	Cabbage Red Acre	sprout	Brassicaceae	*Brassica oleracea*	Cabbage Red Acre Seed	Botanical Interests	74.0
**34**	alyssum	sprout	Brassicaceae	*Lobularia maritima*	Flower Alyssum Sweet Alyssum D1932 (White) 1000 Open Pollinated Seeds by David's Garden Seeds	David's Garden Seeds	14.3
**35**	wasabi	tube paste	Brassicaceae	*Wasabia japonica*	S&B - Wasabi in Plastic Tube (Family Size) 3.17 Oz.	S&B	100.0
**36**	water cress	seed	Brassicaceae	*Nasturtium*	Organic Watercress Seeds - 200 mg -	Botanical	50.0

				officinale	Nasturtium officinale	Interests	
**37**	chinese broccoli	sprout	Brassicaceae	*Brassca Oleracea var.alboglabra*	2000+ Kai Lan Chinese Broccoli Kale Seeds Vegetable Seeds	SeedSeeds	74.0
**38**	curled mustard	sprout	Brassicaceae	*Brassica juncea*	Earthcare Seeds Southern Giant Curled Mustard 6000 Seeds (Brassica juncea)	EarthCare Seeds	13.7
**39**	Horse radish	leaf	Brassicaceae	*Armoracia rusticana*	Horseradish Roots Natural Organic Ready to Plant 1 Pound	Growerssolution	35.0
**40**	Wasabi	sprout	Brassicaceae	*Wasabia japonica*	100pcs/lot Wasabi Seeds, Japanese Horseradish Seed Vegetable Seeds Bonsai Plant DIY Home Garden Plants	chawi chongyang	25.0
**41**	Wasabi	powder	Brassicaceae	*Wasabia japonica*	S&B Wasabi Powder, 1.06 Ounce	S&B	32.0
**42**	karashi	powder	Brassicaceae	*Brassica juncea*	S&B Mustard Powder, 3-Ounce	S&B	100.0
**43**	wild mustard	stem	Brassicaceae	*Sinapis arvensis*	Harvested in California	-	49.3
**44**	wild mustard	leaf	Brassicaceae	*Sinapis arvensis*	Harvested in California	-	6.2
**45**	wild mustard	flower	Brassicaceae	*Sinapis arvensis*	Harvested in California	-	7.8
**46**	Arugla	root	Brassicaceae	*Eruca vesicaria*	Produced & harvested in Davis, California	-	50.0
**47**	Arugla	stem	Brassicaceae	*Eruca vesicaria*	Produced & harvested in Davis, California	-	16.0
**48**	Arugla	leaf	Brassicaceae	*Eruca vesicaria*	Produced & harvested in Davis, California	-	5.6
**49**	water cress	leaf	Brassicaceae	*Nasturtium officinale*	Product of California	Nugget Market	7.0
**50**	water cress	stem	Brassicaceae	*Nasturtium officinale*	Product of California	Nugget Market	13.6
