# Supplementary material for: Occurrence of urea-based soluble epoxide hydrolase inhibitors from the plants in the order Brassicales
Source: PLoS One. 2017 May 4;12(5):e0176571. doi: 10.1371/journal.pone.0176571 (PMC5417501; doi:10.1371/journal.pone.0176571)
Supplement: S5 Table — (PDF) [file pone.0176571.s007.pdf]

S5 Table. Relative potency of reverse phase-HPLC fractions

HPLC fraction	sEH inhibitory potency recovery % relative to crude extract[a]
12-14 min	3
22-24 min	4
24-26 min (contains compound **8**)	5
26-28 min	3
28-30 min	3
30-32 min	5
32-34 min	7
34-36 min	8
36-38 min	9
38-40 min	9
40-42 min	7
42-44 min	4
Total inhibition activity recovered in fractions above	68
Crude extract (filtered & evaporated)	100

[a]The sEH inhibitory potency recovery percentage from the crude extract was calculated by the formula: Recovery % = Potency of the fraction/Potency of Crude extract × 100.
