# Supplementary material for: Occurrence of urea-based soluble epoxide hydrolase inhibitors from the plants in the order Brassicales
Source: PLoS One. 2017 May 4;12(5):e0176571. doi: 10.1371/journal.pone.0176571 (PMC5417501; doi:10.1371/journal.pone.0176571)

Figure S1a.

^{1}H NMR (DMSO-d_6 with 0.03% v/v TMS, 800MHz)
Compound **1** synthetic standard

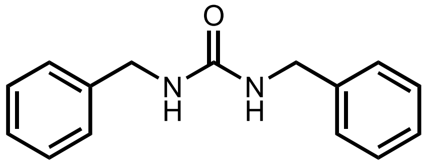

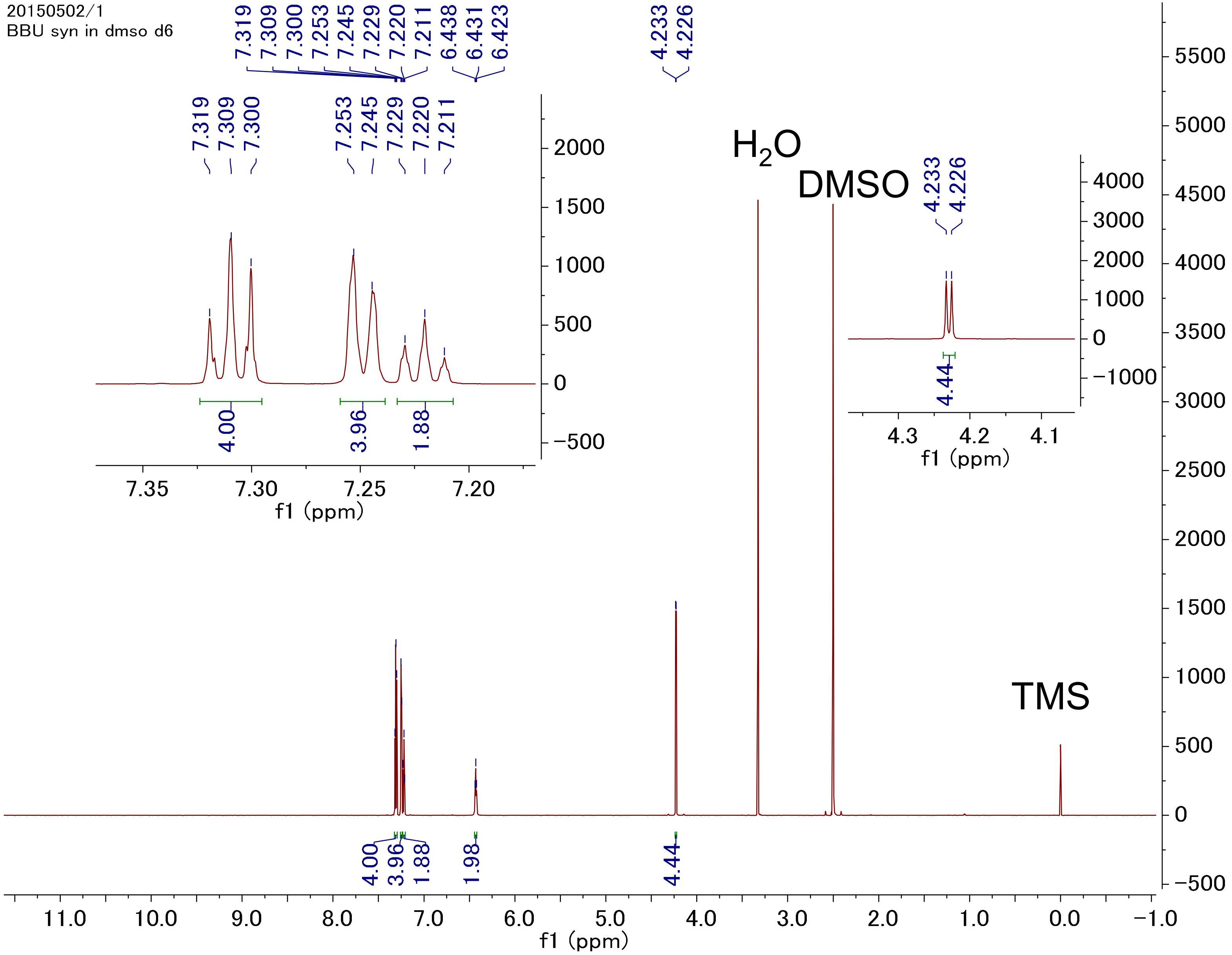

Figure S1b.

^{1}H NMR (DMSO-d_6 with 0.03% v/v TMS, 800MHz)
Compound **1** Isolated
from maca root

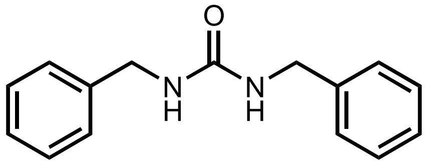

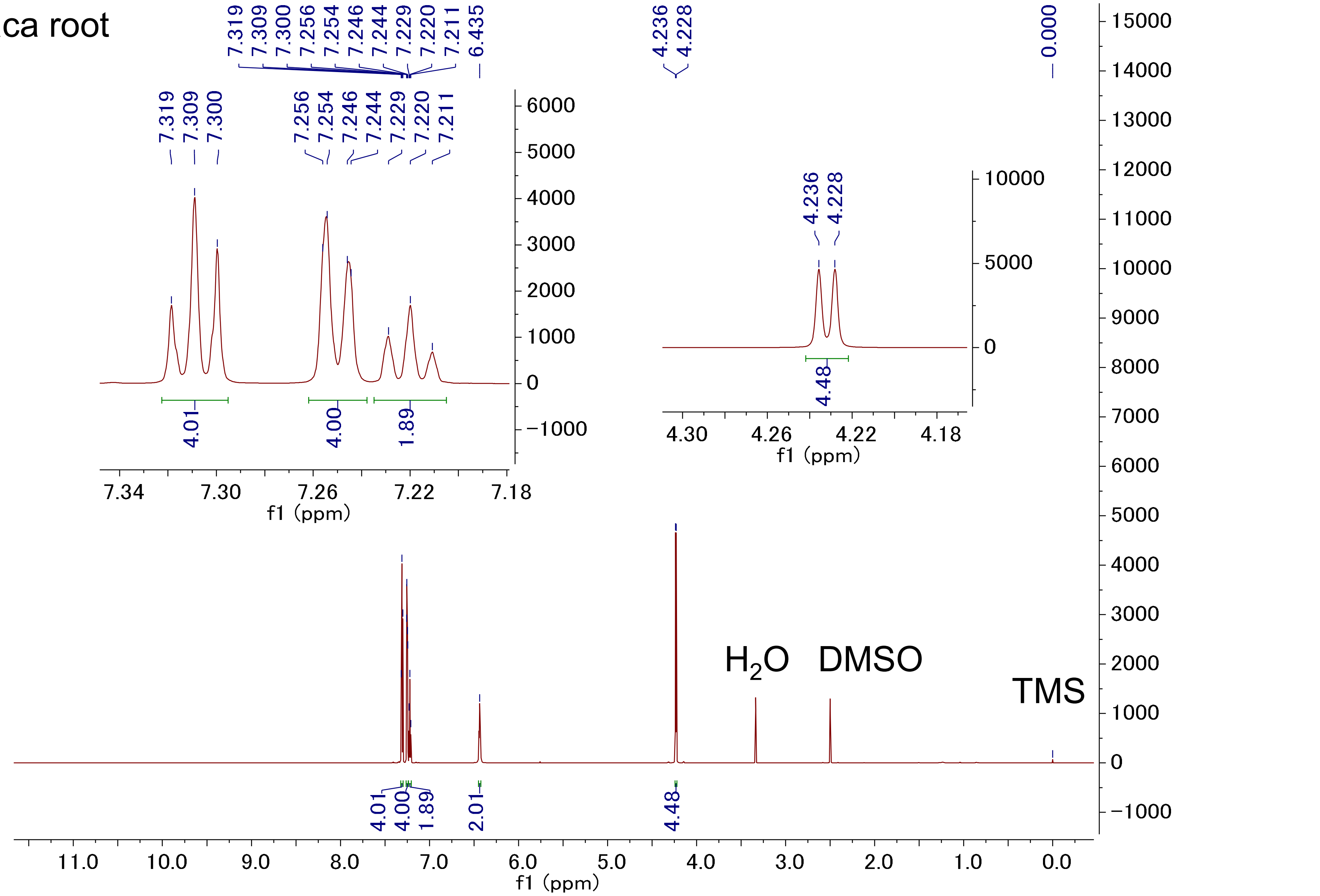

Figure S1c.
^{13}C NMR (DMSO-d_6 with 0.03% v/v TMS, 201MHz)
Compound **1** from maca root

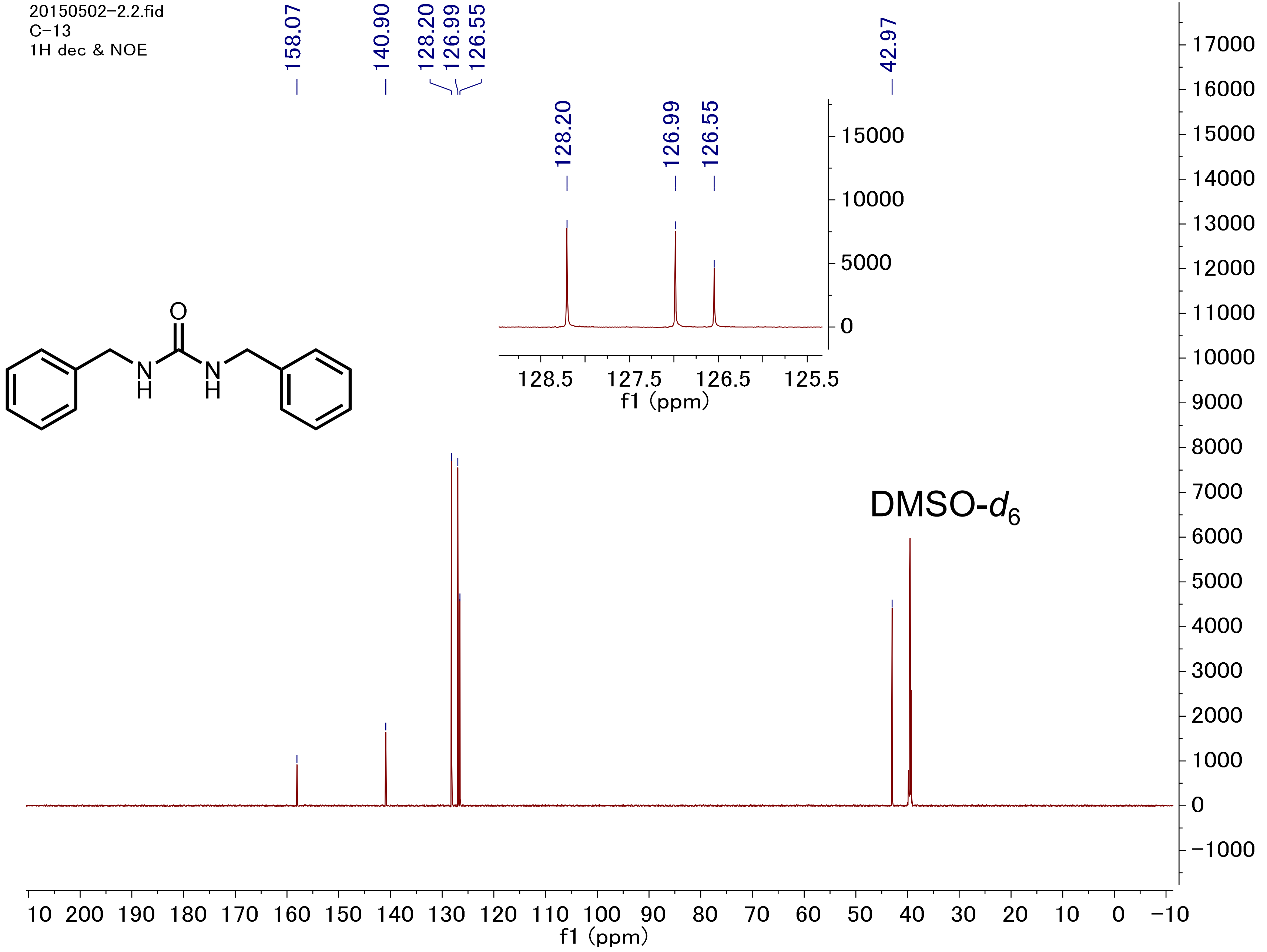

Supplement: S1 Fig — (PDF) [file pone.0176571.s008.pdf]
