# Supplementary material for: Occurrence of urea-based soluble epoxide hydrolase inhibitors from the plants in the order Brassicales
Source: PLoS One. 2017 May 4;12(5):e0176571. doi: 10.1371/journal.pone.0176571 (PMC5417501; doi:10.1371/journal.pone.0176571)

Figure S2a.
^{1}H NMR (DMSO-d_6 with 0.03% v/v TMS, 600MHz)
Synthetic standard (compound **2**)

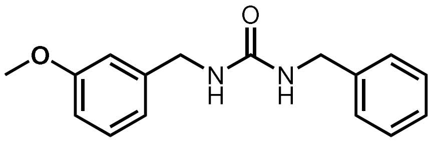

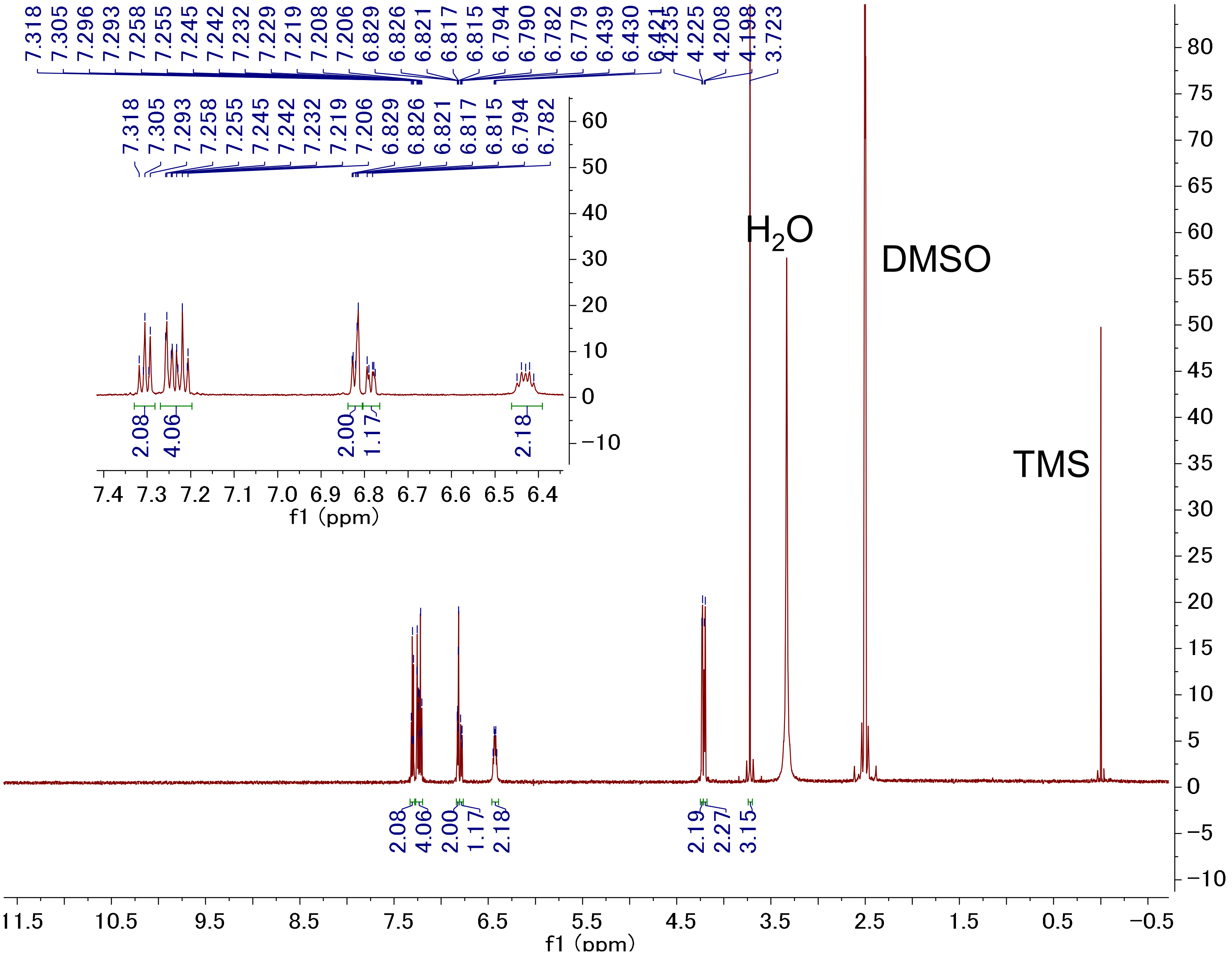

Figure S2b.
^{1}H NMR (DMSO-d_6 with 0.03% v/v TMS, 800MHz)
Compound **2** from maca root

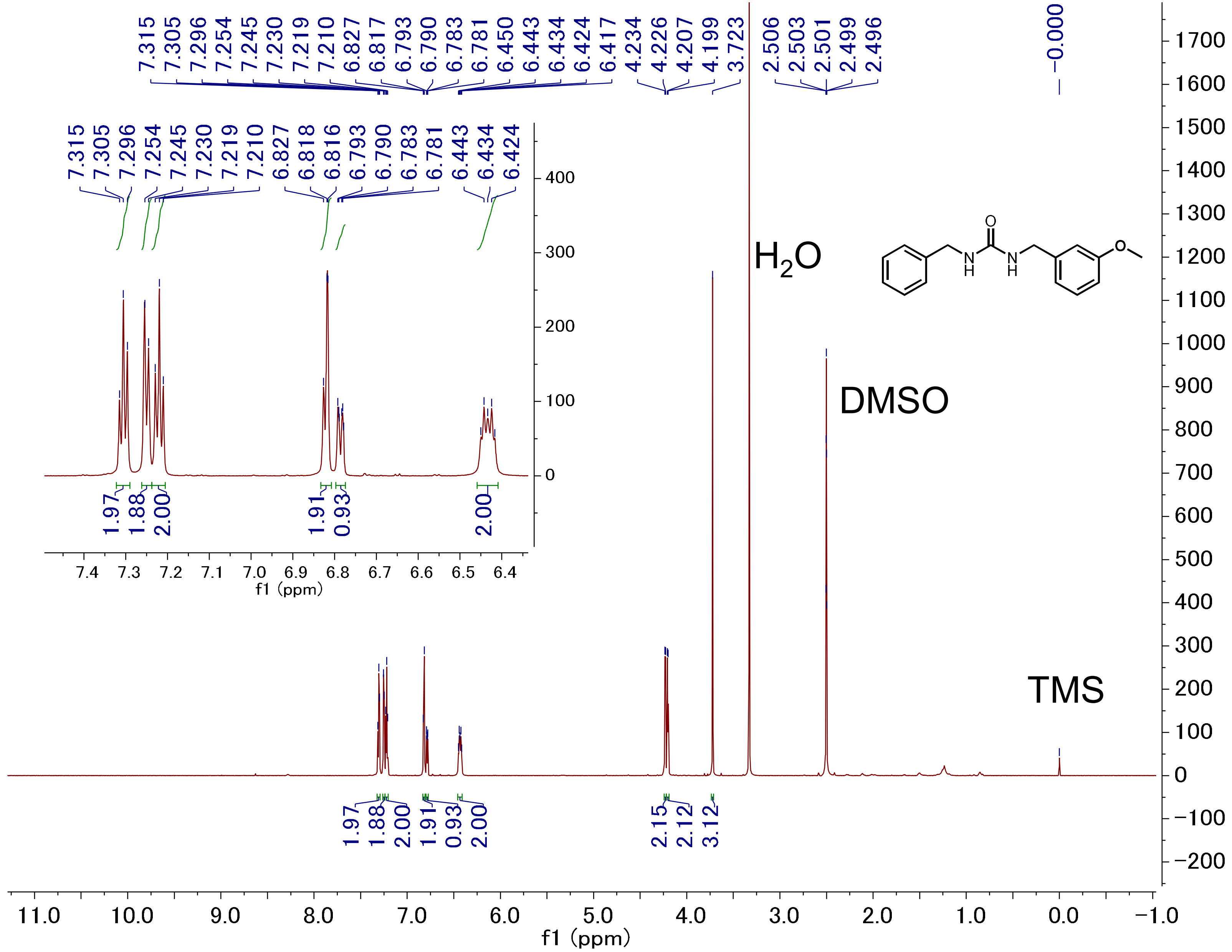

Figure S2c.

^{13}C NMR (DMSO-d_6 with 0.03% v/v TMS, 201MHz)

Compound **2** from maca root

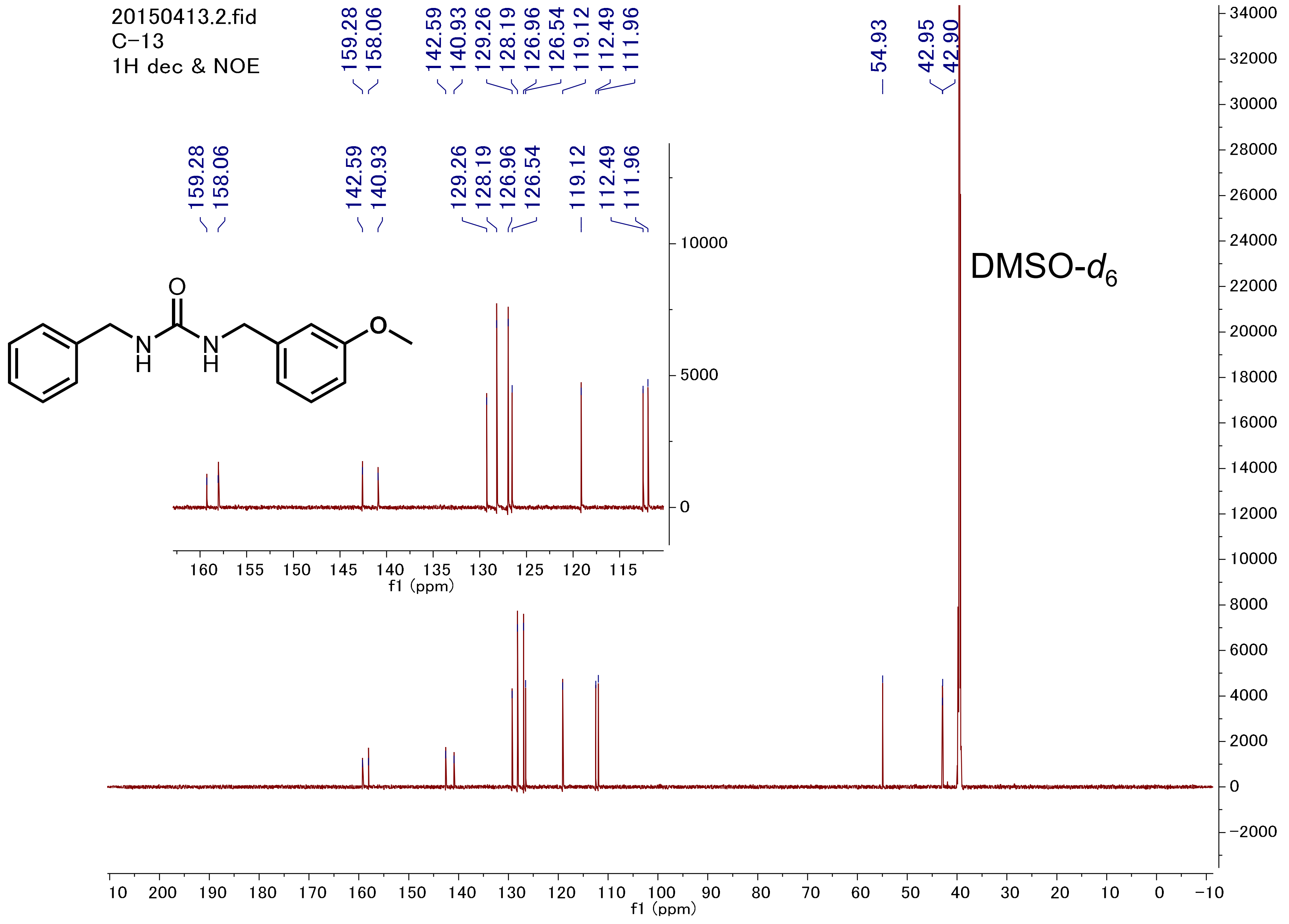

Supplement: S2 Fig — (PDF) [file pone.0176571.s009.pdf]
