# Supplementary material for: Occurrence of urea-based soluble epoxide hydrolase inhibitors from the plants in the order Brassicales
Source: PLoS One. 2017 May 4;12(5):e0176571. doi: 10.1371/journal.pone.0176571 (PMC5417501; doi:10.1371/journal.pone.0176571)

Figure S3a.
^{1}H NMR ($CDCl_3$ with 0.03% v/v TMS, 600MHz)
Synthetic standard (compound **3**)

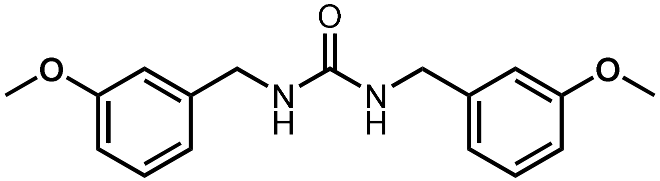

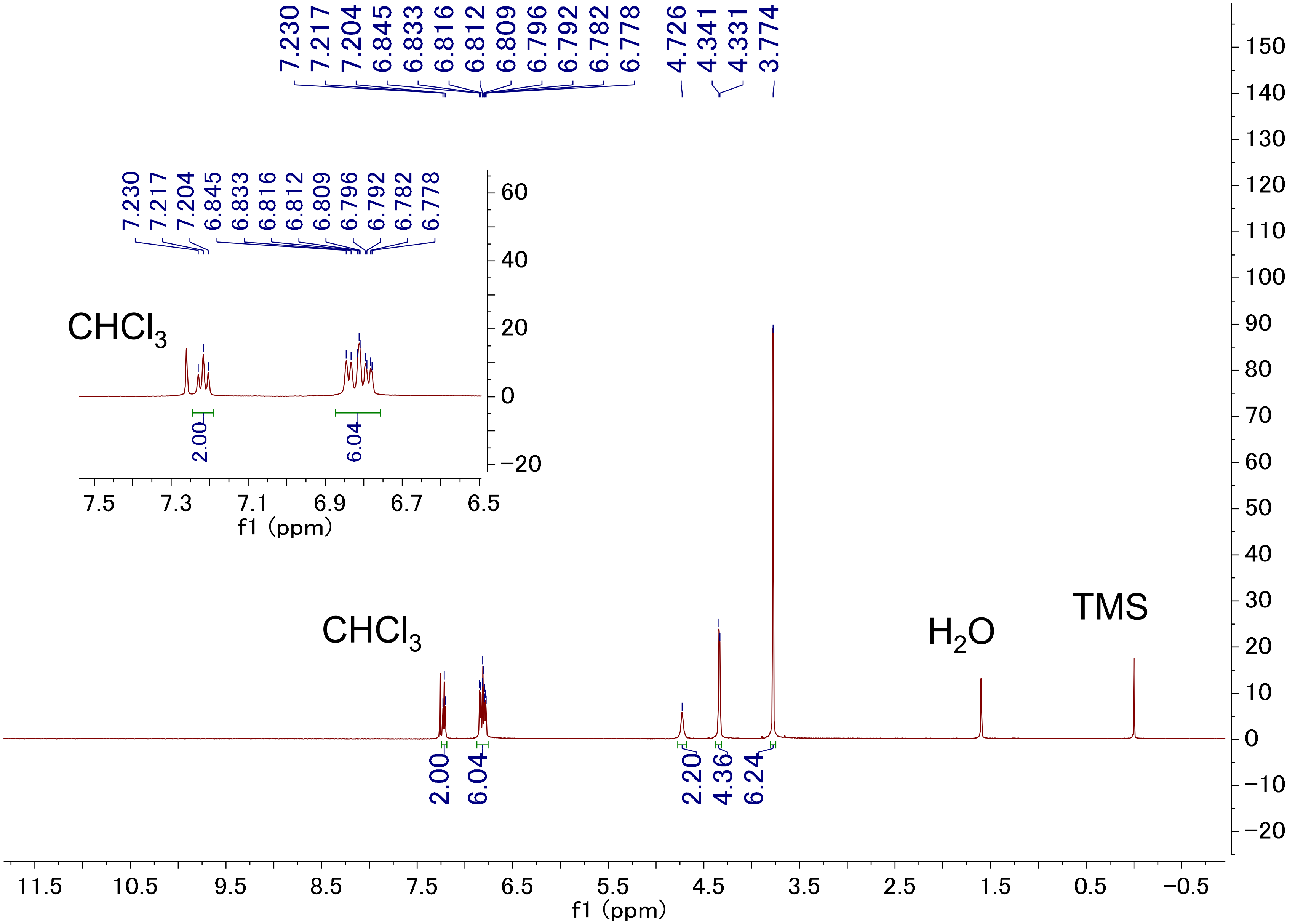

Figure S3b.

^{1}H NMR ($CDCl_3$ with 0.03% v/v TMS, 800MHz) Compound **3** from maca root

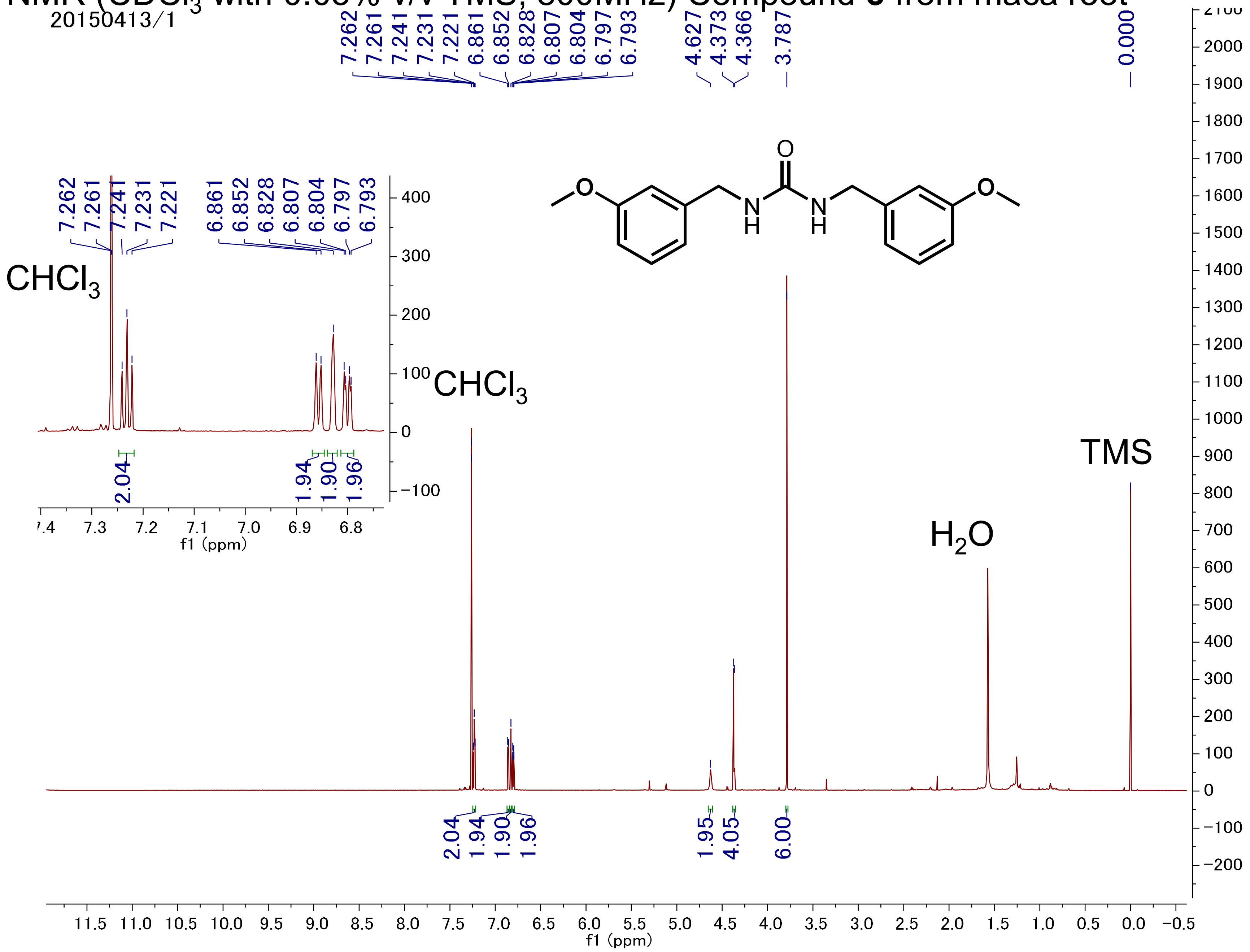

Figure S3c.
^{13}C NMR ($CDCl_3$ with 0.03% v/v TMS, 201MHz)
Compound **3** from maca root

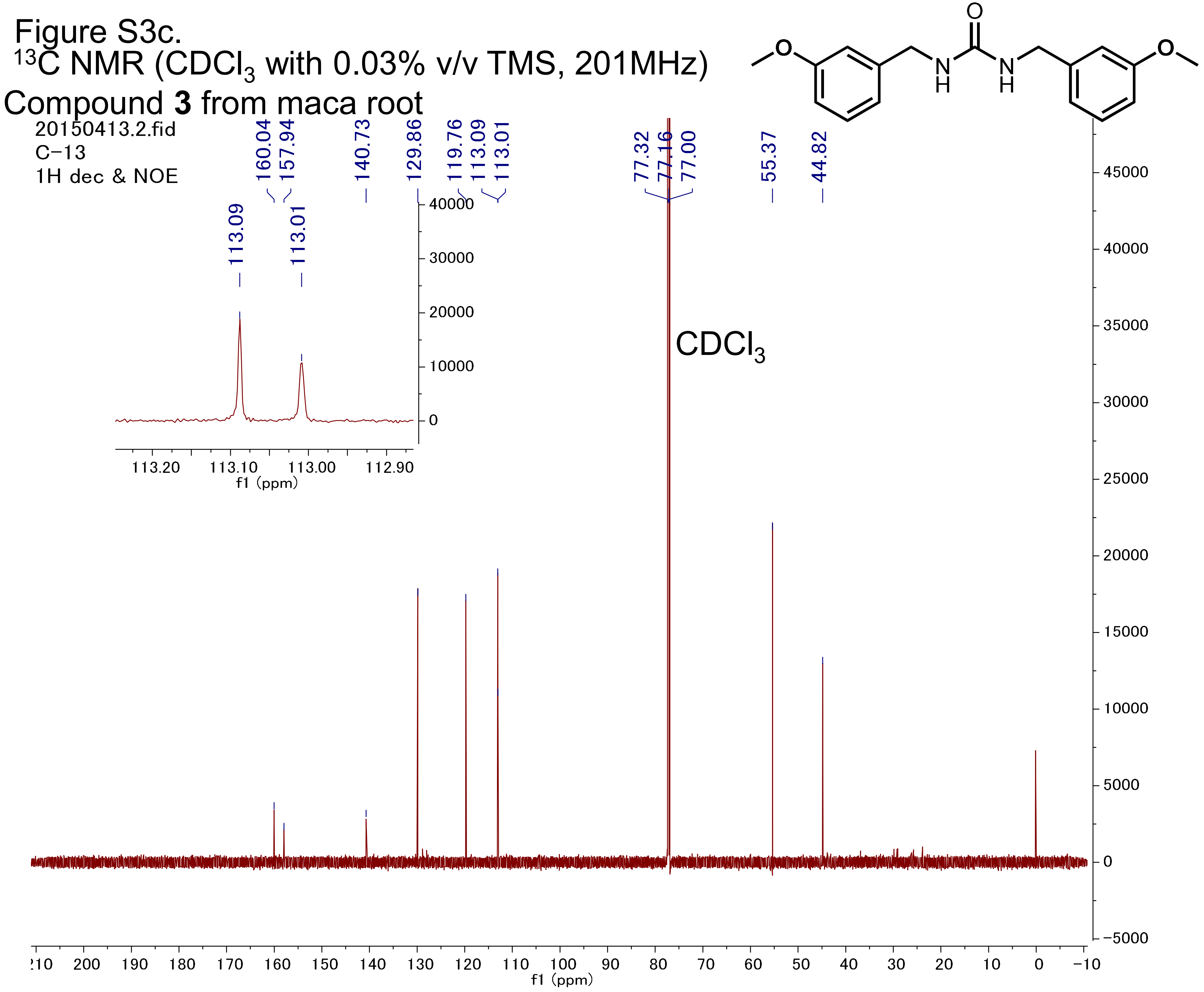

Supplement: S3 Fig — (PDF) [file pone.0176571.s010.pdf]
