# Supplementary material for: Occurrence of urea-based soluble epoxide hydrolase inhibitors from the plants in the order Brassicales
Source: PLoS One. 2017 May 4;12(5):e0176571. doi: 10.1371/journal.pone.0176571 (PMC5417501; doi:10.1371/journal.pone.0176571)

Figure S5. HRESIMS spectra of compound **2** isolated from maca

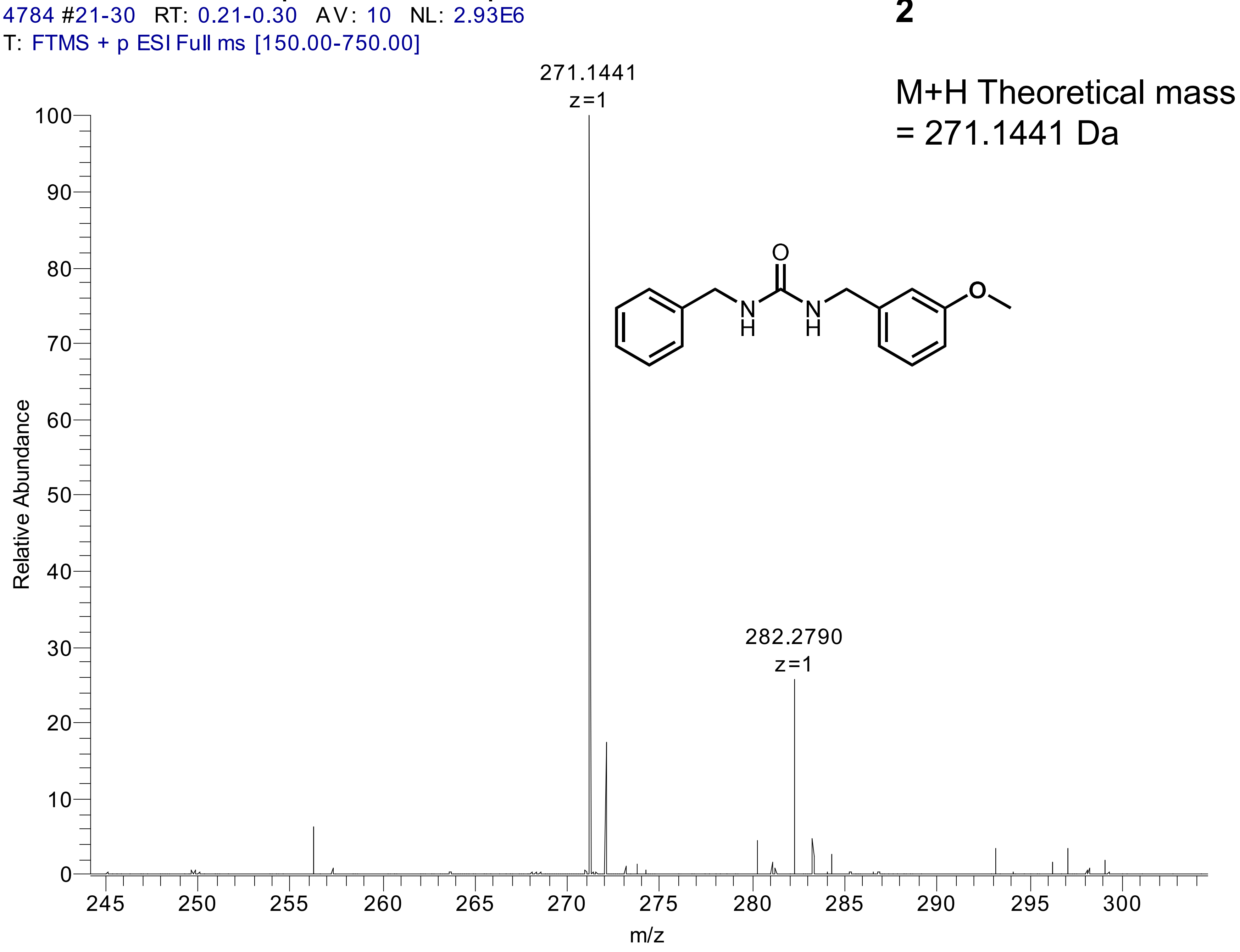

Supplement: S5 Fig — (PDF) [file pone.0176571.s012.pdf]
