# Supplementary material for: Occurrence of urea-based soluble epoxide hydrolase inhibitors from the plants in the order Brassicales
Source: PLoS One. 2017 May 4;12(5):e0176571. doi: 10.1371/journal.pone.0176571 (PMC5417501; doi:10.1371/journal.pone.0176571)
Supplement: S7 Fig — (PDF) [file pone.0176571.s014.pdf]

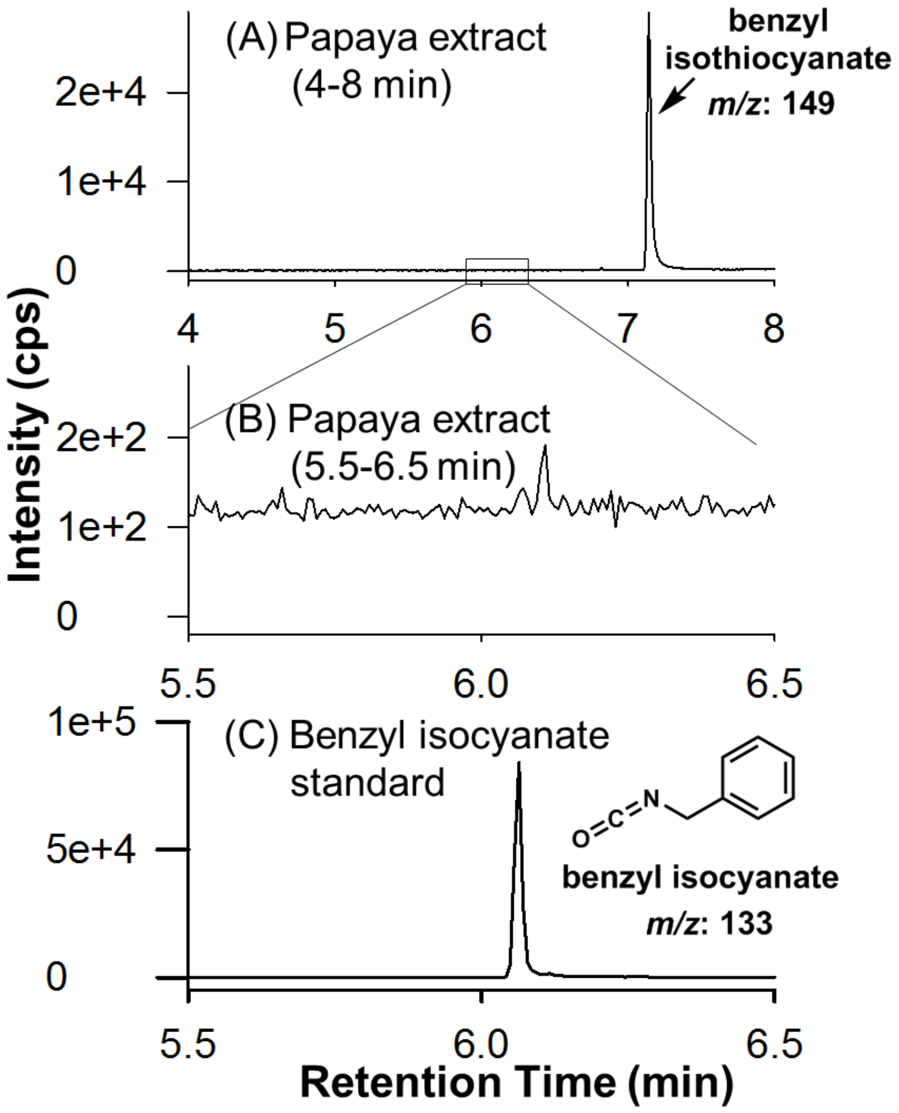

S7 Fig. GC-MS analysis of benzyl isocyanate and benzyl isothiocyanate. Cps: counts per second. (A-C) Selected ion monitoring (SIM) chromatogram analyzing *m/z*=133 (benzyl isocyanate) and 149 (benzyl isothiocyanate). Benzyl isocyanate and benzyl isothiocyanate had retention times at 6.05 min and 7.15 min, respectively. (A) Papaya seed extract (5 µg fresh seed/µL). Retention time 4-8 min is shown. (B) Papaya seed extract (5 µg fresh seed/µL). Retention time 5.5-6.5 min is shown. (C) Benzyl isocyanate standard (1 µg/mL).

Fresh papaya seeds (2 g) were ground and incubated 10 sec at RT, then extracted with dichloromethane (DCM) by sonication (4 mL) at RT (10 sec). The DCM soluble fraction was filtered, then diluted into appropriate concentration in DCM for the GC-MS analysis.

Compounds were analyzed using a Hewlett-Packard 7890 gas chromatograph equipped with an Agilent Technologies 5975B Mass Spectral Detector. DB5MS column (L-30 m, 0.25 mm i.d. film 0.25 mm, Agilent, Santa Clara, CA) was used to separate the analytes. Helium was used as a carrier gas (Airgas, Sacramento, CA). Injection (1 µL) was splitless with the injection port at 150 ºC. Flow rate was 1.0 mL/min. The temperature program had an initial temperature of 30 ºC which was held for 2.25 min. Temperature was ramped at 40 ºC/min to 300 ºC which was held for 3 min. The total run time was 12.00 min. MS was performed in electron impact ionization mode in scanning mode and single ion monitoring (SIM) mode. SIM acquisition included *m/z* 133 and 149 for benzyl isocyanate and benzyl isothiocyanate, respectively.
