# Supplementary material for: Occurrence of urea-based soluble epoxide hydrolase inhibitors from the plants in the order Brassicales
Source: PLoS One. 2017 May 4;12(5):e0176571. doi: 10.1371/journal.pone.0176571 (PMC5417501; doi:10.1371/journal.pone.0176571)
Supplement: S8 Fig — (PDF) [file pone.0176571.s015.pdf]

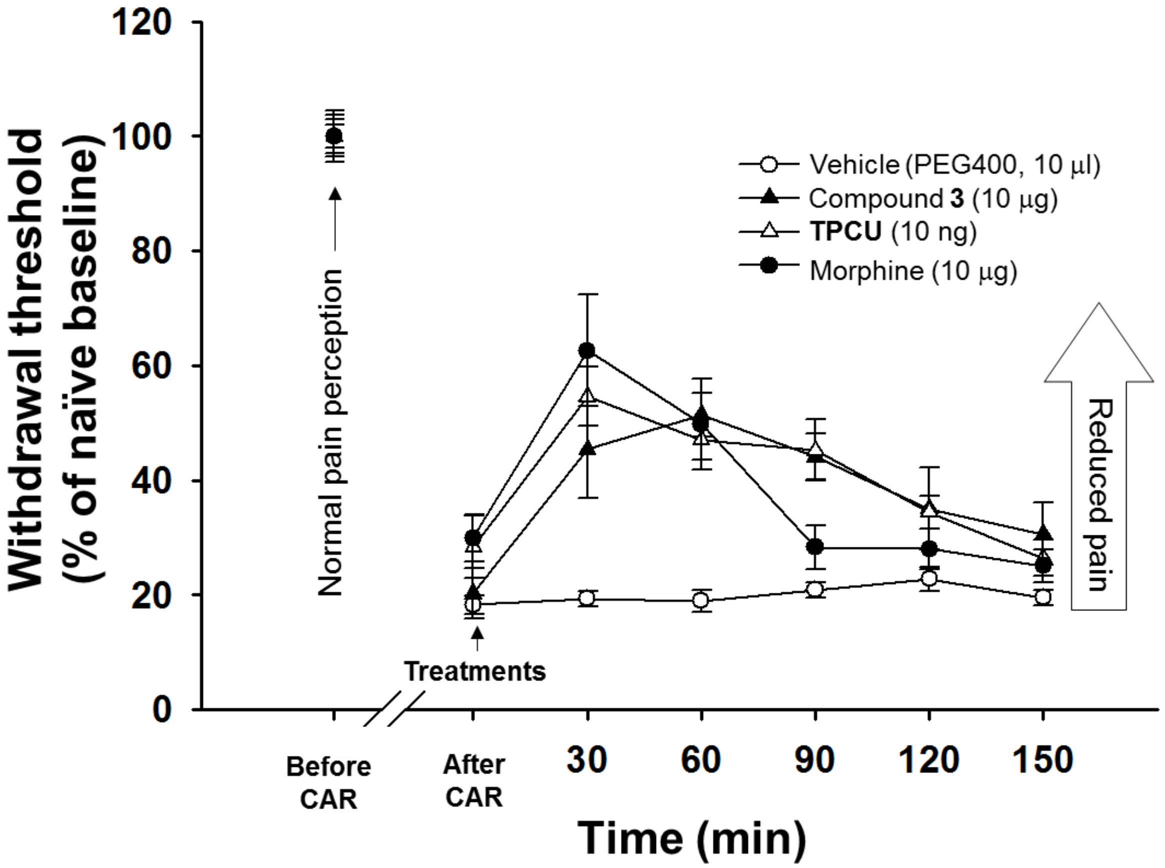

S8 Fig. **Intraplantar administration of compound 3 effectively reduces carrageenan-induced inflammatory pain in rat.** Administration of the inflammatory agent carrageenan (CAR) induces a stable hyperalgesic response for the duration of the experiment. Treatment with **3** (▲, 10 µg/paw, intraplantar), morphine (●, 10 µg/paw, intraplantar) or the potent synthetic sEH inhibitor (1-(1-(Cyclopropanecarbonyl) piperidin-4-yl)-3-(4-(trifluoromethoxy) phenyl) urea, TPCU) (△, 10 ng/paw, intraplantar) significantly reduced pain levels (Kruskal-Wallis One Way ANOVA on Ranks, $p \leq 0.001$, Tukey's post hoc test (compound **3** vs. vehicle, morphine vs. vehicle, TPCU vs. vehicle, $p < 0.05$). Mean ± SE ($n=6$) of mechanical withdrawal threshold (% of naïve baseline) are shown. Vehicle (○), morphine (●), and TPCU (△) treatment data are from Rose *et al*., (2010).
